# Disentangling age‐ and disease‐related alterations in schizophrenia brain network using structural equation modeling: A graph theoretical study based on minimum spanning tree

**DOI:** 10.1002/hbm.25403

**Published:** 2021-05-07

**Authors:** Xinyu Liu, Hang Yang, Benjamin Becker, Xiaoqi Huang, Cheng Luo, Chun Meng, Bharat Biswal

**Affiliations:** ^1^ The Clinical Hospital of Chengdu Brain Science Institute, MOE Key Laboratory for Neuroinformation University of Electronic Science and Technology of China Chengdu China; ^2^ Center for Information in Medicine, School of Life Science and Technology University of Electronic Science and Technology of China Chengdu China; ^3^ Glasgow College University of Electronic Science and Technology of China Chengdu China; ^4^ Huaxi MR Research Center (HMRRC), Department of Radiology West China Hospital of Sichuan University Chengdu China; ^5^ Department of Biomedical Engineering New Jersey Institute of Technology Newark New Jersey USA

**Keywords:** graph theory, mediation analysis, minimum spanning tree, resting‐state FMRI, schizophrenia, structural equation modeling

## Abstract

Functional brain networks have been shown to undergo fundamental changes associated with aging or schizophrenia. However, the mechanism of how these factors exert influences jointly or interactively on brain networks remains elusive. A unified recognition of connectomic alteration patterns was also hampered by heterogeneities in network construction and thresholding methods. Recently, an unbiased network representation method regardless of network thresholding, so called minimal spanning tree algorithm, has been applied to study the critical skeleton of the brain network. In this study, we aimed to use minimum spanning tree (MST) as an unbiased network reconstruction and employed structural equation modeling (SEM) to unravel intertwined relationships among multiple phenotypic and connectomic variables in schizophrenia. First, we examined global and local brain network properties in 40 healthy subjects and 40 schizophrenic patients aged 21–55 using resting‐state functional magnetic resonance imaging (rs‐fMRI). Global network alterations are measured by graph theoretical metrics of MSTs and a connectivity‐transitivity two‐dimensional approach was proposed to characterize nodal roles. We found that networks of schizophrenic patients exhibited a more star‐like global structure compared to controls, indicating excessive integration, and a loss of regional transitivity in the dorsal frontal cortex (corrected *p* <.05). Regional analysis of MST network topology revealed that schizophrenia patients had more network hubs in frontal regions, which may be linked to the “overloading” hypothesis. Furthermore, using SEM, we found that the level of MST integration mediated the influence of age on negative symptom severity (indirect effect 95% CI [0.026, 0.449]). These findings highlighted an altered network skeleton in schizophrenia and suggested that aging‐related enhancement of network integration may undermine functional specialization of distinct neural systems and result in aggravated schizophrenic symptoms.

## Graphical abstract

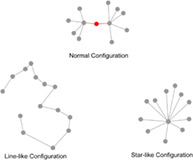

## 1 INTRODUCTION

The brain is a complex system composed of structurally and functionally interacting regions. Modeling intricate interactions of neuronal populations using network languages has demonstrated that brain regions are interconnected in a manner that seeks a balance between functional integration and segregation, termed as small‐worldness (Bassett & Bullmore, 2006; Bullmore & Sporns, 2012). Efficient information processing in complex systems requires a topological organization that balances functional integration, which refers to convergent information processing in distributed brain regions, and functional segregation, which refers to region‐specific selective processing. The fine‐grained trade‐off between integration and segregation changes throughout the lifespan (Cao et al., 2014; Damoiseaux, 2017; Smit, De Geus, Boersma, Boomsma, & Stam, 2016) and aberrant topological organization is widely reported in various neuropsychiatric disorders (Bassett & Bullmore, 2006). While most previous studies focused on the influence of a single factor for example, age or disease, several recent studies suggest an interplay between these factors in neuropsychiatric disorders characterized by marked network‐level dysregulations, particularly schizophrenia (Sheffield & Barch, 2016; Sheffield, Rogers, Blackford, Heckers, & Woodward, 2019). However, the exact mechanism underlying possible interactions between aging and disease‐related network disruptions remained unexplored.

In addition to the possible interplay between multiple behavioral and neurological variables, another key consideration when investigating brain‐behavior associations is the choice of different network construction methods. For example, evident discrepancies have been reported on the level of global topological organization in the disease of schizophrenia, with both enhanced integration and segregation of functional brain networks being reported (van den Heuvel & Fornito, 2014). The discrepant findings in the previous literature may reflect that schizophrenia is a highly heterogeneous disorder, or alternatively may reflect the application of heterogeneous approaches for network construction and comparison. For example, network level analyses have employed weighted or binary networks, different parcellation schemes, and different normalization procedures (van Wijk, Stam, & Daffertshofer, 2010). These variances may pose a challenge to a unified recognition of network alteration patterns in the disease.

In this study, we aim to employ a set of graph‐theoretical and statistical tools to help address the above‐mentioned two problems in a clinical cohort of schizophrenia patients. First, we used minimum spanning tree (MST) algorithm as an unbiased network representation method. An MST is a connected subgraph with minimum cost from the original network. Comparisons can then be made based on MSTs constructed in different individuals, thus avoiding arbitrary thresholding or normalization protocols employed in conventional graph theoretical studies. It has been demonstrated that MST is critical to global network communication (Van Mieghem & Magdalena, 2005; Van Mieghem & Van Langen, 2005; Wang, Hernandez, & Van Mieghem, 2008) and the disruption of MST structure has been found in various neuropsychiatric disorders (Stam et al., 2014), including in two schizophrenia electroencephalography (EEG) studies (Jonak, Krukow, Jonak, Grochowski, & Karakuła‐Juchnowicz, 2019; Krukow, Jonak, Karpiński, & Karakuła‐Juchnowicz, 2019). However, to the best of our knowledge, there was no previous study, which applied the MST approach, together with rs‐fMRI, to disentangle network‐level dysregulations in schizophrenia.

In addition to investigating organizational principles in the global network structure using MST, the characterization of distinct roles of individual brain regions in the network is of critical importance to determine brain‐based biomarkers for schizophrenia and to identify targets for novel neuromodulatory treatments that aim at modulating network level disruptions in psychiatric disorders, for example, (Zhao et al., 2019). Two kinds of prominent nodes emerged in previous brain network studies: hubs and connectors (Sporns & Betzel, 2016). A hub is a node with high number of links to other nodes, while a connector serves as a “bridge” between different modules. These two concepts were often mixed and referred to as “connector hubs” (A. F. Alexander‐Bloch et al., 2013; Brandl et al., 2018; Sporns & Betzel, 2016), because nodes with high centrality would also likely play an important role in network transportation. Previous analysis based mainly on centrality may not capture the full spectrum of nodal properties in networks, as it has been found that some low‐degree nodes in biological networks might also be crucial in intermodule communication (Del Ferraro et al., 2018; Joy, Brock, Ingber, & Huang, 2005). We propose that it is more appropriate to delineate nodal roles in MST based on their “connectivity” and “transitivity,” that is, differentiating the ability of serving as a hub or an intermodule connector, where modules are identified as different hubs since clusters are absent in MST. Under the proposed heuristic two‐dimensional framework, we aimed to investigate how the nodal‐level characteristics in MST were disrupted in schizophrenia.

Secondly, to model the interactions among these variables simultaneously, we employed a mediation analysis framework based on structural equation modeling (SEM) (Kline, 2005). This statistical framework allows to identify interwoven causal pathways among multiple variables, and specifically focuses on the mediation effect, where an intervening variable critically mediates the relationship between an independent variable and an outcome variable (Baron & Kenny, 1986; MacKinnon, 2008). Previous studies have demonstrated developmental and age‐related changes in MST integration, such that MST integration increases with age from childhood to adulthood, that is, toward star‐like configurations (Smit et al., 2016). In older populations, aging was found to be related to a loss of segregation of distinct functional systems (Cao et al., 2014; Ferreira et al., 2016; Geerligs, Renken, Saliasi, Maurits, & Lorist, 2015). These two trajectories can be jointly quantified by MST parameters such as leaf fraction. In MST‐based pathology studies, it has been demonstrated that the topology of MSTs is associated with ADHD symptoms (Y. Wang et al., 2019), cognitive impairment in multiple sclerosis (Tewarie et al., 2014), and illness duration in schizophrenia (Jonak et al., 2019). In the present study, we aimed to develop a unified model, which accounts for the intricate interactions between aging, brain network architecture, and behavior performances simultaneously. Based on previous research, we specifically aimed at determining whether alterations in the optimal structure of brain networks, reflected by MST metrics, was influenced by age throughout adulthood and whether these alterations play a role in cognitive impairments and clinical symptoms in schizophrenia.

**FIGURE 1:**
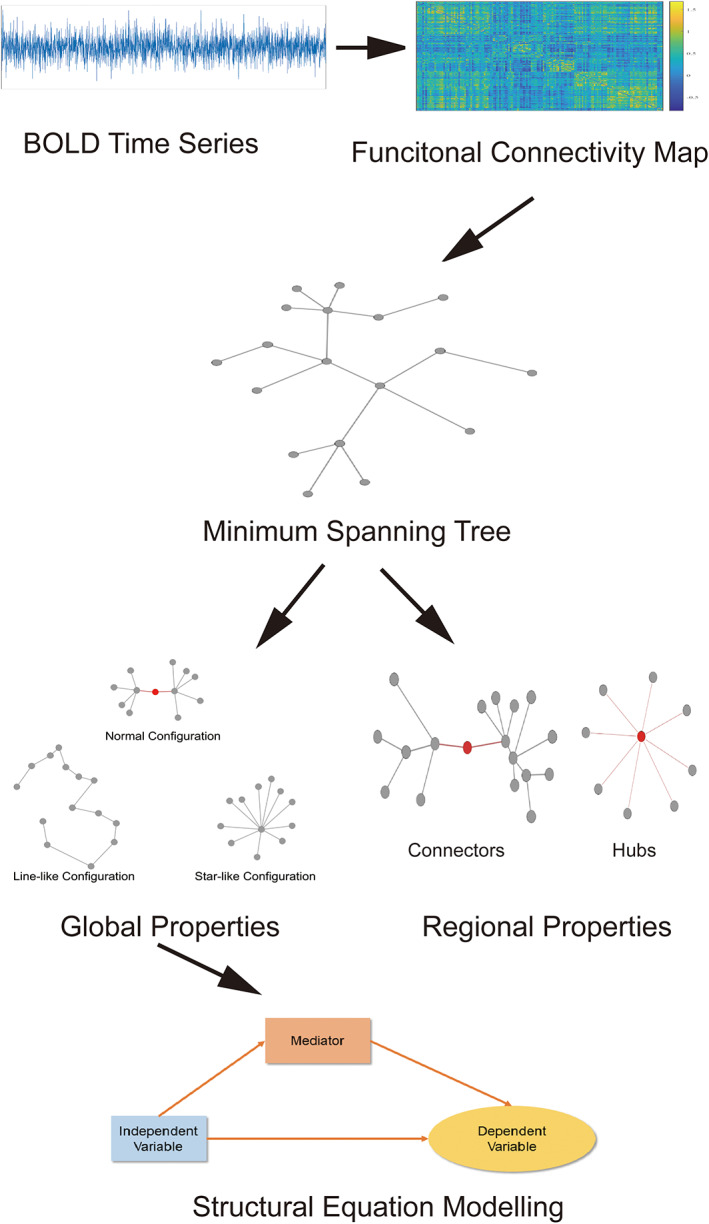
Overview of the analysis flow in the present study. First, we construct brain connectivity map from interdependencies between BOLD signals measured in resting state. Next, the minimum spanning tree algorithm was used to extract the critical skeleton of the brain network. We then studied global network properties reflected by MST metrics. In the meantime, we used the proposed connector‐hub classification scheme to analyze nodal roles. Finally, the global MST metrics were entered into the mediation model to test the hypothesis that the age‐behavior relationship was mediated by brain network structures

In summary, we examined functional brain network changes as well as their interaction with aging and cognitive outcomes using MST algorithm and rs‐fMRI data from 40 schizophrenia patients (22–50 years) and 40 matched healthy control subjects. We tested three hypotheses for the MST: (1) The regular pattern of network integration or segregation observed in healthy controls would be dysregulated in schizophrenia; (2) Regional level properties, characterized by connectivity and transitivity, would be disrupted in schizophrenia; (3) The influence of age on cognitive functions and clinical symptoms would be partly mediated by MST structure change (Figure 1).

## 2 MATERIALS AND METHODS

### 2.1 Participants

The data used in this study is from the UCLA Consortium for Neuropsychiatric Phenomics (CNP) dataset, which is publicly available in the OpenfMRI database with accession number ds000030. A detailed description of the dataset can be found in (Poldrack et al., 2016). All participants gave written informed consent according to procedures approved by the Institutional Review Boards at UCLA and the Los Angeles County Department of Mental Health. Initially, the dataset includes 130 healthy subjects and 50 schizophrenia patients. During preprocessing, subjects were excluded if they had missing structural data, mis‐registration between fMRI and sMRI scans, >2 mm (translation) or 2° (rotation) maximum framewise displacement in the fMRI scan or failure in fMRI normalization. Data from 98 healthy subjects and 40 patients adhered to our quality control process. To address the problem of unbalanced sample size, we used an unbiased bipartite matching method and obtained a sample of 40 healthy and 40 schizophrenia patients matched by age and gender (Figure S1).

Functional MRI data were collected using a T2*‐weighted echoplanar imaging (EPI) sequence with the following parameters: slice thickness = 4 mm, 34 slices, TR = 2 s, TE = 30 ms, flip angle = 90°, matrix 64 × 64, FOV = 192 mm, oblique slice orientation. The resting fMRI scan lasted 304 s, resulting in 152 images for each subject. The parameters for the T1 weighted structural image were the following: TR = 1900 ms, TE = 2.26 ms, FOV = 250 mm, matrix = 256 × 256, sagittal plane, slice thickness = 1 mm, slice number = 176.

### 2.2 Behavioral assessment and dimensionality reduction

All subjects in the present study completed extensive neuropsychological behavior assessments. In the present study, we focused on two cognitive dimensions: executive function and working memory, both of which were reported to be significantly and chronically altered in schizophrenia (Sheffield & Barch, 2016). Briefly, working memory encompasses the ability to store and retrieve information on short time scales, whereas executive functions often encompass a broad range of functions that promote cognitive control and guide behavior. For the patients, we adopted previously established framework for classifying schizophrenia symptoms, in which symptoms were divided into “positive symptoms,” such as delusions hallucinations and disorganization, and “negative symptoms,” including anhedonia, social withdrawals and flattened affect (Sheffield & Barch, 2016). The severity of these symptoms was quantified using Scale for the Assessment of Negative and Positive Symptoms (SANS/SAPS) ratings.

Given that we were concerned with multiple cognitive domains, each of which is reflected by several different assessments, a reduction of dimensionality is vital to account for exponentially increasing model complexity. In SEM, factor analysis is often performed to extract latent variables underlying multiple manifest variables (scores from behavior tests in our case). In our study, for the cognitive tests, the latent cognitive variables were reflected by scores from multiple behavior tests in the same manner performed in a previous research (Kebets et al., 2019). In detail, the score of working memory was built on results of (1) WMS (Wechsler Memory Scale) digital span test; (2) WMS symbol span test; (3) WMS letter‐number sequencing test. Similarly, executive function was based on (1) D‐KEFS (Delis‐Kaplan Executive Function Systems) verbal fluency test; (2) CPT (Continuous Performance Test)‐D prime test; (3) Stroop cognitive conflict test.

For the symptoms, since the factor structure of SANS/SAPS scores is ambiguous and controversial (Andreasen, Arndt, Alliger, Miller, & Flaum, 1995; Emsley, Rabinowitz, Torreman, Early, The RIS‐INT‐35 Early Psychosis Global Working Group, 2003), we conducted an exploratory factor analysis (EFA) to identify the underlying factors. The EFA or CFA (confirmatory factor analysis) represents a common first step in SEM, where the factor loadings would be later integrated into the model. The extracted latent factors broadly agree with the validated three‐factor structure (i.e., paranoia, disorganization, and negative symptoms). Demographics and scores for behavior assessments are summarized in Table 1, where antipsychotics dosage was converted into chlorpromazine‐equivalent value by using ratios presented in (Kroken, Johnsen, Ruud, Wentzel‐Larsen, & Jørgensen, 2009; Leucht et al., 2014). In total, five behavioral domains, including three clinical and two behavioral variables were included in the subsequent correlation and SEM analysis.

**TABLE 1:** Demographics and behavior data for subjects

	Healthy controls (*n* = 40)	Schizophrenia patients (*n* = 40)	HC vs. SZ
			*p* value
Age, mean (*SD*)	37.1 (8.85)	37.4 (8.92)	.892
Male/female	29/11	29/11	1
Antipsychotics dosage, mean (*SD*)	N/A	357.8 (1865.0)	N/A
Working memory, mean (*SD*)	62.2 (11.2)	50.2 (10.8)	<.001
Executive function, mean (*SD*)	249.6 (9.1)	240.2 (9.38)	<.001
Scale for the Assessment of Negative Symptoms (SANS), mean (*SD*)	N/A	1.44 (0.72)	N/A
Framewise displacement (translation), mean	0.4019	0.5763	<.001
Framewise displacement (rotation), mean	0.3713	0.3700	.985
Scale for the Assessment of Positive Symptoms (SAPS), mean (*SD*)	N/A	0.63 (0.44)	N/A
Working memory tests			
WMS Digital Span	28.0 (6.3)	23.0 (4.9)	<.001
WMS Symbol Span	23.8 (6.7)	17.3 (6.4)	<.001
WMS Letter‐Number Sequencing	19.8 (2.5)	17.4 (3.3)	<.001
Executive function tests			
D‐KEFS Verbal Fluency	39.9 (10.8)	30.0 (8.5)	<.001
CPT‐D Prime	322.0 (6.3)	317.5 (11.7)	.04
Stroop Conflict Effect	0.98 (0.03)	0.96 (0.05)	.05

### 2.3 FMRI data preprocessing

The fMRI data were preprocessed in MATLAB using SPM8 (http://www.fil.ion.ucl.ac.uk/spm) and Data Processing & Analysis for Brain Imaging (DPABI) tool (Yan, Wang, Zuo, & Zang, 2016). The following preprocessing procedures were performed: (1) Removal of the first 2 time points (Di & Biswal, 2018; Kebets et al., 2019; Mellem et al., 2020); (2) Realignment to adjust head motion; (3) co‐Registration to the structural T1 image; (4) Normalization by using T1 segmented DARTEL (Ashburner, 2007); (5) Nuisance signal regression, including 24 head motion parameters, white matter signal and cerebrospinal fluid signal (Hallquist, Hwang, & Luna, 2013); (6) Linear detrending; (7) Band‐pass filtering of 0.01–0.1 Hz; (8) Smoothing with an 8 mm FWHM kernel.

In addition, to test the validity of our results, we performed a series of validation analysis in which processing strategies were varied and the results were examined. Details of our validation studies were presented in Section 2.6.3.

### 2.4 Network construction

We chose Dosenbach's 160‐ROI parcellation scheme (Dosenbach et al., 2010) to construct functional brain networks. According to the previous study (Di & Biswal, 2018), bilateral amygdala and parahippocampus are not covered by Donsenbach's 160 ROIs, and need be added, in order to cover all of typical subcortical areas, resulting in 164 cortical and subcortical ROIs covering the whole brain. The edges of the network were defined as Fisher's Z‐score transformed correlation coefficients of ROI‐specific time series. As a result, a 164*164 adjacency matrix representing the network for each subject was obtained.

### 2.5 Graph theoretical analysis

#### 2.5.1 MST generation

The MST of an undirected weighted network is an acyclic subgraph connecting all nodes with minimized overall weight. The MST is unique provided that the weights are unique in the original network, which ensures us to circumvent the need of thresholding and inclusion of spurious weak edges. Though there are alternative methods to handle negative edges in graph theoretic analysis (Schwarz & McGonigle, 2011), in current study we chose to remove negative edges before the MST generation because of the requirement of MST algorithm and to be consistent with previous studies (Stam et al., 2014; Tewarie, van Dellen, Hillebrand, & Stam, 2015; van Montfort et al., 2018). In addition, since we are concerned with strongest connections in brain network analysis, we converted the connection weight matrix to distance matrix using $d_{\mathrm{ij}}=\frac{1}{w_{\mathrm{ij}}}$ prior to subsequent analysis. Kruskal's algorithm (Kruskal, 1956) is then applied to extract the MST. Briefly, the algorithm starts by removing all edges in the graph and then adds edges one by one while ensuring the overall weight is minimized and avoiding formation of loops in the graph. The use of MST allows us to analyze the critical skeleton of the network, which could be potentially useful to gain new insight in the pathology of the disease.

#### 2.5.2 Similarity analysis

To quantify the similarity between MSTs, we simply calculated the pairwise overlap (Lee & Kim, 2006):

$$\sigma \left(a,b\right)=\frac{1}{n-1}\left|E_{a}⋂E_{b}\right|,$$

where *n* is the total number of nodes in an MST. In the equation, *E*_*a*_ and *E*_*b*_ refer to the set of edges of two MSTs *a* and *b* respectively, ⋂ takes the intersection of two sets, and |·| denotes the cardinality of a set. Thus, the overlap (or survival ratio) calculates how many edges two MSTs have in common and thus can be a measure of similarity.

We then performed a nonparametric permutation test to examine whether there is a difference in terms of network structure between two groups. A similar approach was used to detect whether the network community structure of schizophrenia patients differs from healthy subjects (A. Alexander‐Bloch et al., 2012). First, the group membership is randomized for each subject, followed by a comparison between the actual and permuted within‐group similarity. The within‐group similarity is defined as the average value of all pairwise overlaps within a group. If there was a true difference between the original two groups (schizophrenia and healthy control) in terms of MST structure, the randomly permutated within‐group similarity should be consistently lower than the true within‐group similarity in most permutations. Otherwise, there should be no difference in network structure between the two groups. Note that, we do not need to calculate between‐group similarity in this approach, since we are only concerned with actual and permutated within‐group similarity. The *p* value is set as the number of times that permuted within‐group similarity is not less than the actual within‐group overlap divided by 10,000 times of permutations in total.

#### 2.5.3 Global level analysis

To characterize the global structure of the MST, we calculated path length, leaf fraction, tree hierarchy, maximum degree, assortativity, and degree divergence for each subject. Definitions and explanations of these metrics are summarized in Table 2. (Tewarie et al., 2015).

**TABLE 2:** Symbols and definitions of MST metrics

Symbol	Variable name	Definition
L	Path length	L is the average shortest path length between any pair of nodes in a network.
Lf	Leaf fraction	Lf is the fraction of nodes with degree 1. Higher Lf indicates more star‐like, integrated network; low Lf on the other hand indicates greater level of segregation.
Th	Tree hierarchy index	Quantifies the trade‐off between integration in the MST and overloading. Th = 0.5 implies star‐like topology, Th = 1 signifies line‐like structure.
*D*_max_	Maximum degree	The maximum value of degree in the network.
*r*	Assortativity	Quantifies the tendency of nodes to link to other nodes with similar degrees.
*κ*	Degree divergence	Measure of the broadness of the degree distribution.

**FIGURE 2:**
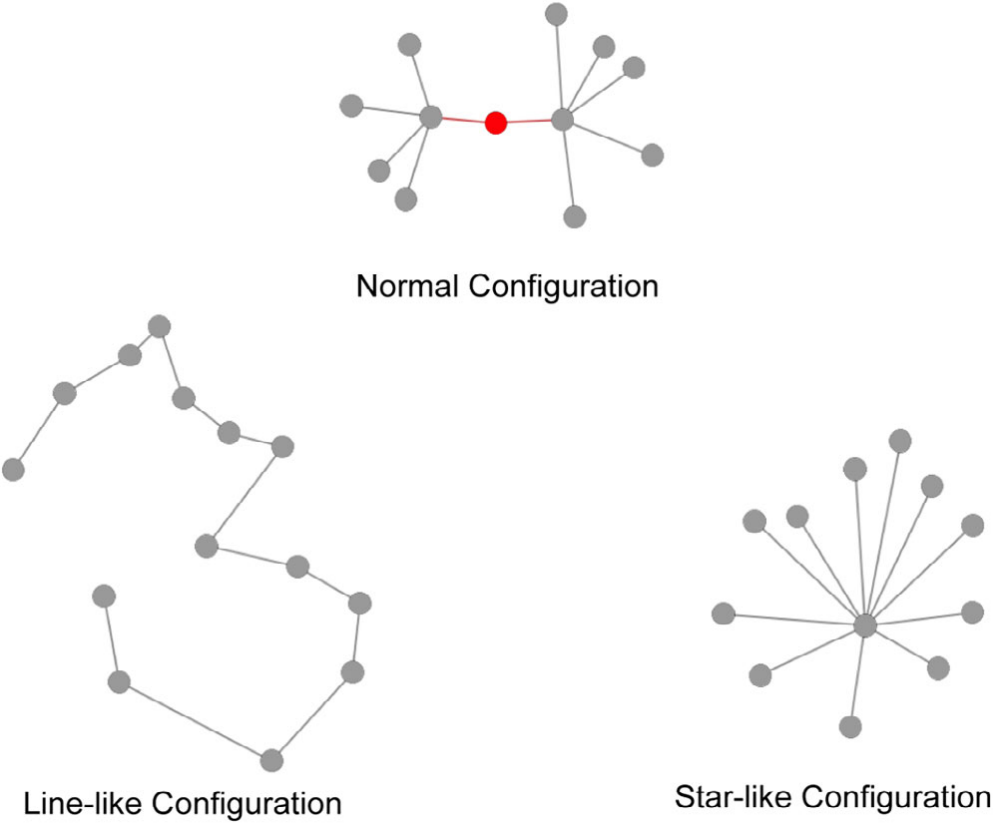
Different minimum spanning tree global organizations. On the top is the hypothesized normal structure which should be expected to be seen in healthy adults. The state is an intermediate configuration between two extremes. When nodes become increasingly segregated, the tree would transform into the line‐like structure in the lower‐left corner; on the other hand, the star‐like structure represent highly centralized network arrangement

The metrics we calculated can be a multifaceted statistical representation of different MST configurations. Path length is a classical statistic used in conventional network studies, which reflects the integration of network, and higher degree divergence signifies the presence of more hubs (thus more low‐degree nodes). In addition, higher leaf fraction and maximum degree indicates a tree containing more star‐like local structures, thus being more integrated. On the other hand, lower leaf fraction implies a trend toward line‐like network, thus increased segregation. A schematic illustration of different MST configurations is shown in Figure 2.

#### 2.5.4 Regional level analysis

In traditional analysis of networks, degree and betweenness centrality were often used to measure node importance. A heuristic demonstration of nodal roles was shown in Figure 2, where the node in red with only degree 2 is critical for the communication between two relatively high‐degree hubs. Therefore, there are two kinds of nodes of high betweenness: hubs and “bridges.” The present study aims to propose a classification scheme to distinguish hubs and intermodule connectors.

Although the use of MST precludes the presence of densely connected community structures, different high‐degree hubs can be thought of as independent functional units in such a sparse network. A star‐like structure is one which consists of one central node (hub) and several peripheral nodes (leaves) connected to the central one with only one link. The interconnection of multiple stars forms a tree. According to the definition of communities as internally dense and externally sparse sub‐graphs, each star within one general tree naturally forms one cluster, because in each star most leaves exclusively link to their own hub, except for a small number of leaves connecting the rest of the graph. In the meantime, nodes with relatively lower degree but connecting different hubs are likely critical to global network communication. These phenomena are often ignored in previous MST research where only degree or betweenness are analyzed. To differentiate these “bridges” or connectors from hubs in an MST, we propose a new graph theoretical based statistic to measure the ability of a node to serve as “connectors” rather than hubs.

Definition 1 For any node *v* in a network of *n* nodes in total, The local importance (li) of a node *v* is defined as follows (H. Yu, Jiao, Yao, & Wang, 2016):

$$\mathrm{li}\left(v\right)=\frac{\left|A\right|}{\left|\mathrm{Adj}\left(v\right)\right|}+\frac{1}{n}$$

where *A* = {*u* ∈ *Adj*(*v*)| *Degree*(*u*) ≤ *Degree*(*v*)}. *Adj*(*v*) represents the neighbor of node *v*,and |·| denotes the cardinality, that is, the number of elements, of a set, and n represents the number of nodes in a network. It is assumed that *Adj*(*v*) ≠  ∅ .

In the definition, the set A contains all nodes in the neighbor of *v* whose degree is not greater than *v*. Thus, *li*(*v*) = 1/*n* if all nodes in adjacent to *v* have higher degree than it, and $\mathrm{li}\left(v\right)=1+\frac{1}{n}\;$ if all nodes are of lower degree than *v*. The term 1/*n* can be seen as the “baseline” importance, stemming from the intuition that the more nodes there are in a network, the less important each individual node is. Hence, the value of *li* can quantify the importance of a node in comparison with its neighbors, that is, the local importance.

Definition 2 The connector index (ci) of a node is defined as

$$\mathrm{ci}\left(v\right)=\frac{\mathrm{BC}\left(v\right)}{\mathrm{li}\left(v\right)},$$

where *BC*(*v*) denotes the betweenness of *v*. By this definition, a node would have a high *ci* if they are responsible for greater amount of globally integrated traffic (high BC) and are locally unimportant. In contrary, hub nodes would have low ci because of their high local importance. Thus, *ci* can be an appropriate measure of the ability of serving as “bridges” rather than hubs in an MST.

Connector index and degree can form a two‐dimensional “connectivity–transitivity” framework to characterize nodal roles in an MST, analogous to the notion of *z* − *P* parameter space proposed for general networks (Guimerà, 2005; van den Heuvel & Sporns, 2011). High‐degree nodes can be seen as hubs, while high‐ *ci* nodes are important bridges which account for the majority of communication. Nodes with both low degree and *ci* are less important peripheral regions. This classification would help us better understand roles of individual nodes in the global brain network, and aberrant values of these indexes could also signal dysfunction of corresponding regions, particularly in integrating information from neighborhood regions or facilitate communication among different regions, in psychiatric diseases like schizophrenia.

In our study, we choose group‐representative MST to conduct identification of spatial distribution of hubs/connectors and following analysis of degree distribution. This can be derived by first averaging connection matrix within each group, and then applying the MST algorithm on the group‐averaged connectivity matrix. It is a common practice to evaluate nodal roles in group‐averaged networks to reduce complexity (Brandl et al., 2018). Since a group‐averaged matrix may be influenced by outliers, we evaluated the mean and standard deviation of average within‐group similarity of each subject. We found that the healthy group has a mean of 0.267 and standard deviation of 0.201, the schizophrenia group has a mean of 0.250 and standard deviation of 0.191. With fairly low standard deviation, it was therefore reasonable to represent each group using group‐averaged matrix. We denote nodes with degree of 2 *SD* higher than the mean value of all nodes as hubs, and nodes with *ci* of 2 *SD* higher than the mean value as connectors seeking a balance between significance and number of important nodes (L. Wang, Metzak, Honer, & Woodward, 2010).

#### 2.5.5 Degree distribution analysis

We then explored possible degree distribution patterns in the group‐level MST. Degree distribution is a concept originated from network science, and was extensively used to investigate the global organization patterns of the brain network (E. T. Bullmore & Bassett, 2011). Briefly, a power‐law distribution indicates that the brain contains a small subset of regions which have extensive connections to other regions (i.e., hubs) and are likely to play important roles in the brain's functional integration. On the contrary, in a random graph the degree distribution may follow a Poisson distribution where there are rarely nodes with significantly more connections. Thus, the analysis of degree distribution can reveal a global pattern of inter‐region cooperation in the brain. The degree distribution of brain networks was initially thought to be close to a power‐law distribution or scale‐free structure of the form *P*(*k*) = *k* ^−*α*^, where *P*(*k*) indicates the number of nodes that have the degree of k. However, later evidence suggested that brain network may instead exhibit an exponentially truncated power law distribution of the form $P\left(k\right)=k^{\alpha -1}e^{k/k_{c}}$, in which the probability of high‐degree nodes will be higher than in a random graph (exponential degree distribution *P*(*k*) = *e* ^−*αk*^) but smaller than in a scale‐free network (Achard, Salvador, Whitcher, Suckling, & Bullmore, 2006; Bassett & Bullmore, 2006). To test the degree distribution obtained from MST networks, we fitted the group‐level distribution to the three distributions. The goodness of fit was assessed by *R*‐squared values.

### 2.6 Statistical analysis

#### 2.6.1 Correlation between variables

It has been shown that MST global network measures are strongly correlated with characteristic path length and clustering coefficient in the original network (Tewarie et al., 2015). In the present study, we further explored the intercorrelation between each pair of MST metrics using Pearson's correlation coefficient. In general, all MST measures were positively or negatively correlated with each other as expected, because they are indicators of network structure toward integration or segregation (Smit et al., 2016).

To investigate whether there exists any specific association between predictors (age, MST metrics) and outcomes (behavior), we estimated partial correlations between each pair of these variables (Lynall et al., 2010). Generally, partial correlations were found to be nonsignificant, suggesting that no specific relationship between predictors and outcomes can be isolated and therefore there may be more complex interactions among them, as revealed by our structural equation model. Results for correlations between these variables are shown in Supporting Information.

#### 2.6.2 Group differences

We tested group differences in global network metrics listed in Table 2 and local metrics (degree, ci) using nonparametric permutation test of 5,000 permutations with age, gender, and head motion controlled as covariates. In permutation tests, group assignment was randomized to yield an empirical null distribution and the hypothesis that there is no difference between the two groups is tested. False discovery rates (FDR) were corrected using Benjamini and Hochberg procedures (Benjamini & Hochberg, 1995) for 6 global MST metrics and for 164 regions × 2 regional MST metrics, respectively. For regional metrics showing significant difference between groups, we additionally performed an exploratory correlation with the five behavior dimensions to see if the regional aberration could be related to behavior.

#### 2.6.3 Validation analysis

We performed a series of validation studies to examine the robustness of our results across different data processing strategies.

First, It has been found that quantitative measures of network properties may vary across different parcellation schemes (de Reus & van den Heuvel, 2013). To validate the reproducibility of our results, first, we replicated our similarity and global level analysis on three commonly‐used parcellation schemes: (1) Harvard–Oxford 112‐ROI atlas; (2) Power's 264‐ROI atlas (Power et al., 2011); (3) Schaefer's 400‐ROI atlas (Schaefer et al., 2018).

Second, we performed wavelet despiking using the BrainWavelet Toolbox (Patel et al., 2014; Patel & Bullmore, 2016) to further reduce artifacts caused by head motion and replicated results reported in the main text. For the wavelet despiking, a maximum‐overlap discrete wavelet decomposition (MODWT) was applied to the BOLD time series of each voxel. Outlying wavelet coefficients were regarded as noise and discarded, and the remaining coefficients were retained and used for the subsequent construction of the denoised signal using inverse MODWT. The method has been shown to be an effective way to account for head motion related artifacts, superior to many of the traditional censoring or ICA‐based methods (Parkes, Fulcher, Yücel, & Fornito, 2018).

Third, we evaluated the effect of removal of four time points (instead of two) on our results, to ensure better stabilization of the scanner. Last, we performed global signal regression as an additional step in preprocessing to evaluate the effect of global signal on our results.

### 2.7 Structural equation modeling

We hypothesized that increased age would lead to higher level of MST integration (or decreased segregation) during adulthood and thus change behavior outcomes. In other words, we tested a mediation effect (Baron & Kenny, 1986; Hayes, 2009) where the influence of age on clinical or cognitive measures is mediated by brain network structure. Of note, the mediation model itself is based on regression, thus can only make inferential claims about the causal relationships between variables. With this in mind, we build our model on a logical basis, with variables placed in their most reasonable positions (e.g., we hypothesize that increased age would influence brain structure, but it would be obviously invalid in logic to claim that changes in brain would lead to changes of the age of the subject). In this study, we picked leaf fraction as the representative variable for MST structure for model simplicity because of high correlations among different tree metrics, indicating possible redundancy (Table S2). Alternatively, the network structure can be construed as a latent variable estimated from six graph metrics. We evaluated this model construction scheme and presented the results in Supporting Information.

In addition to mediation analysis, we further tested an alternative moderation mechanism that is possibly existing among the variables to examine whether the influence of age on leaf fraction was moderated by schizophrenia (as a status variable). Moderation analysis was used to probe whether the relationship (strength or direction) between two variables was moderated by the third variable. To test the moderation effect, one adds an additional interaction term of age × group into the multiple regression analysis and evaluated the coefficient. It can be concluded that there is a significant moderation effect if the coefficient of the interaction term is significantly nonzero. In total, we tested four models:

Model 1: Mediation model: (age)–(leaf fraction)–(working memory, executive function). The sample only includes 40 healthy individuals.

Model 2: Mediation model: (age)–(leaf fraction)–(working memory, executive function). The sample includes 40 healthy subjects and 40 schizophrenia patients.

Model 3: Mediation model: (age)–(leaf fraction)–(psychotic symptom, negative symptom, disorganization).

The sample includes 40 schizophrenia patients. The three latent variables were based on previously established three‐factor structure (Andreasen et al., 1995).

Model 4: Moderation model: The influence of age on leaf fraction were moderated by schizophrenia (as a categorical variable). The sample includes all 80 subjects.

**FIGURE 3:**
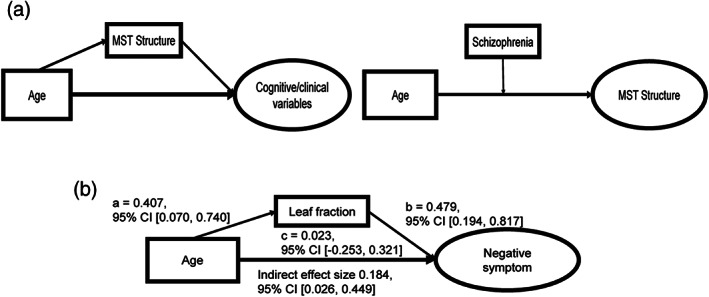
Structural equation models. (a) The two models which could potentially depict interactions between age, network structure and behavior. The first mediation model to the left posits that aging would lead to higher leaf fraction, which represents higher level of integration and decreased segregation, and then induce behavior changes. The moderation model examines whether the relationship between age and network structure is influenced by schizophrenia. (b) Primary parameters of the significant indirect effect between age and negative symptom severity through leaf fraction (CI, credible interval)

A schematic illustration of the proposed models is shown in Figure 3a. All variables were standardized prior to analysis, and we included head motion, gender, and medication use of schizophrenic patients as covariates by specifying them as exogenous predictors of mediators and outcome variables to regress out their possible influence on mediators and behavior. For simplicity, covariates and behavior measures (as manifest variables) are not presented in the figure but an illustration of full models can be found in Figures S9 and S10. SEM analyses were conducted using Mplus 8.1 (L. K. Muthén & Muthén, 2017). Since the sample size is relatively small in the current study, Bayesian estimation with noninformative prior distributions is used for parameter estimation. Bayesian structural equation modeling (BSEM) has been recommended when analyzing relatively small samples because of its independency on large‐sample theory. Similar to other parameter estimation methods used in mediation analysis (i.e., bootstrapping), BSEM does not make assumptions of the parameter distribution in the estimation (B. Muthén & Asparouhov, 2012). In addition, Bayesian method permits unbiased estimation of indirect effects with asymmetric confidence intervals in mediation analysis (B. Muthén & Asparouhov, 2012). Model fit for BSEM is evaluated by the posterior predictive *p*‐value (PPP). The index is analogous to the chi‐square p value in maximum likelihood estimation; values of PPP greater than .05 indicate good model fit (B. Muthén & Asparouhov, 2012).

## 3 RESULTS

### 3.1 Demographic and behavioral result

Demographic and clinical data are presented in Table 1. Healthy individuals did not differ from patients in age and gender. However, as expected, schizophrenic patients obtained significantly lower scores in multiple behavior tests compared to healthy subjects, indicating marked cognitive deficits in schizophrenia. Specifically, schizophrenic patients showed significantly lower performances in all three tests relating to working memory, but only two tests related to executive function, suggesting more pronounced working memory deficits. The pattern of behavioral deficits are in line with cognitive impairments in schizophrenia found in several previous studies (For a review, see (Sheffield & Barch, 2016)).

### 3.2 Similarity and global network organization

**FIGURE 4:**
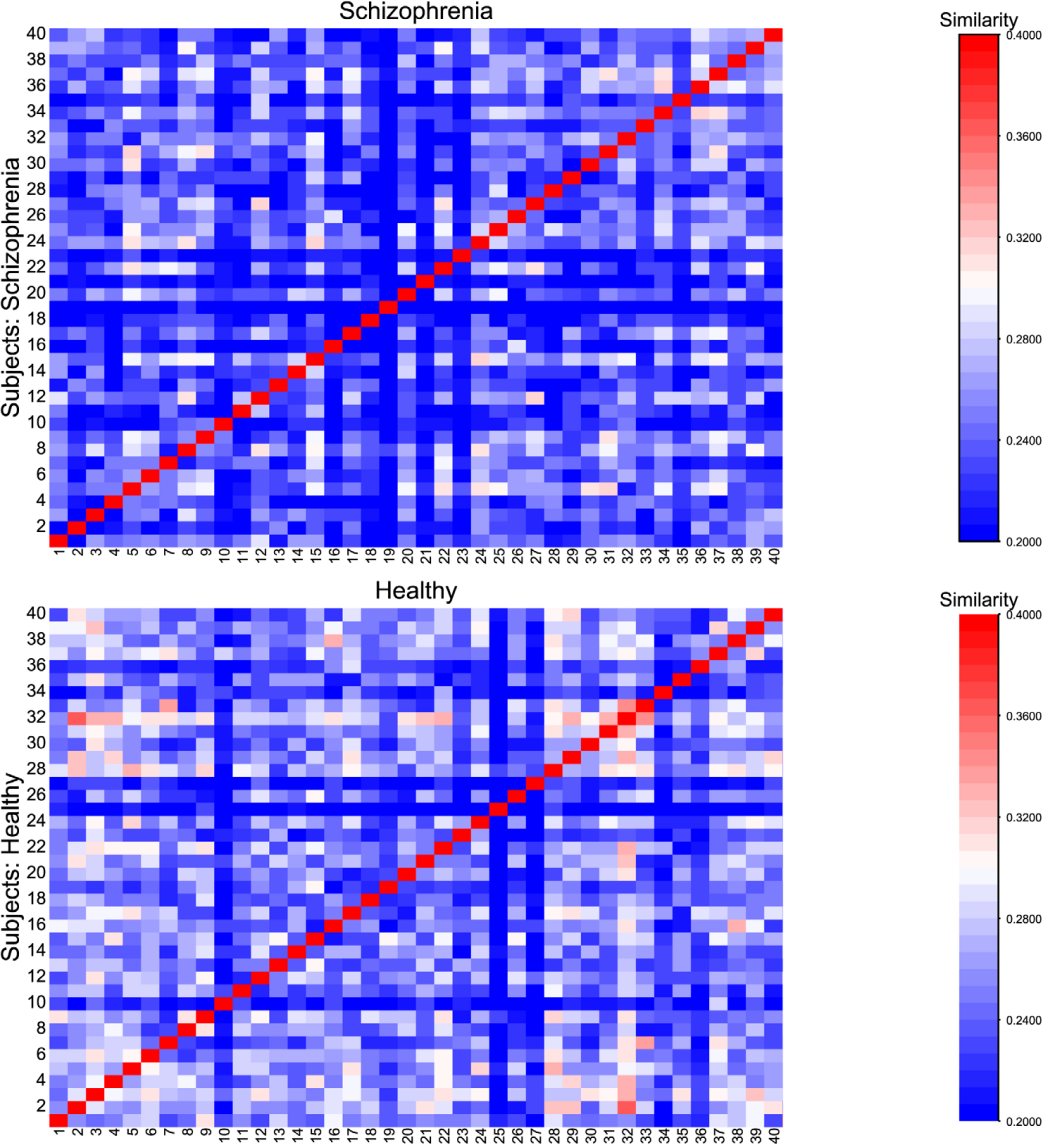
Within‐group similarity matrix for both healthy and schizophrenia groups. Each element is the value of overlapping rate between two subjects

Pairwise overlap between individual MSTs were calculated based on the number of edges they have in common. Based on the measure of similarity, we found novel evidence for an altered MST structure in schizophrenia patients. Figure 4 showed within‐group similarity matrices for both groups. Each row or column represent a subject, and values in each position in the matrix represent the calculated pairwise overlap rate. Averaging the mean value of overlap rate for both matrices yielded a mean within‐group overlap rate of 0.267 for healthy controls and 0.250 for patients. Since the mean value in healthy group is higher than the disease group, intuitively speaking there should be a higher level of heterogeneity in network structure existing in schizophrenia patients.

Permutation test further revealed that the actual within‐group similarity was significantly higher than within‐group similarity generated by randomized group assignment (*p* = .04). Therefore, the actual intragroup overlapping rate was significantly higher than the value that would be expected by chance, suggesting that there was a genuine difference in MST structure between the two groups.

**FIGURE 5:**
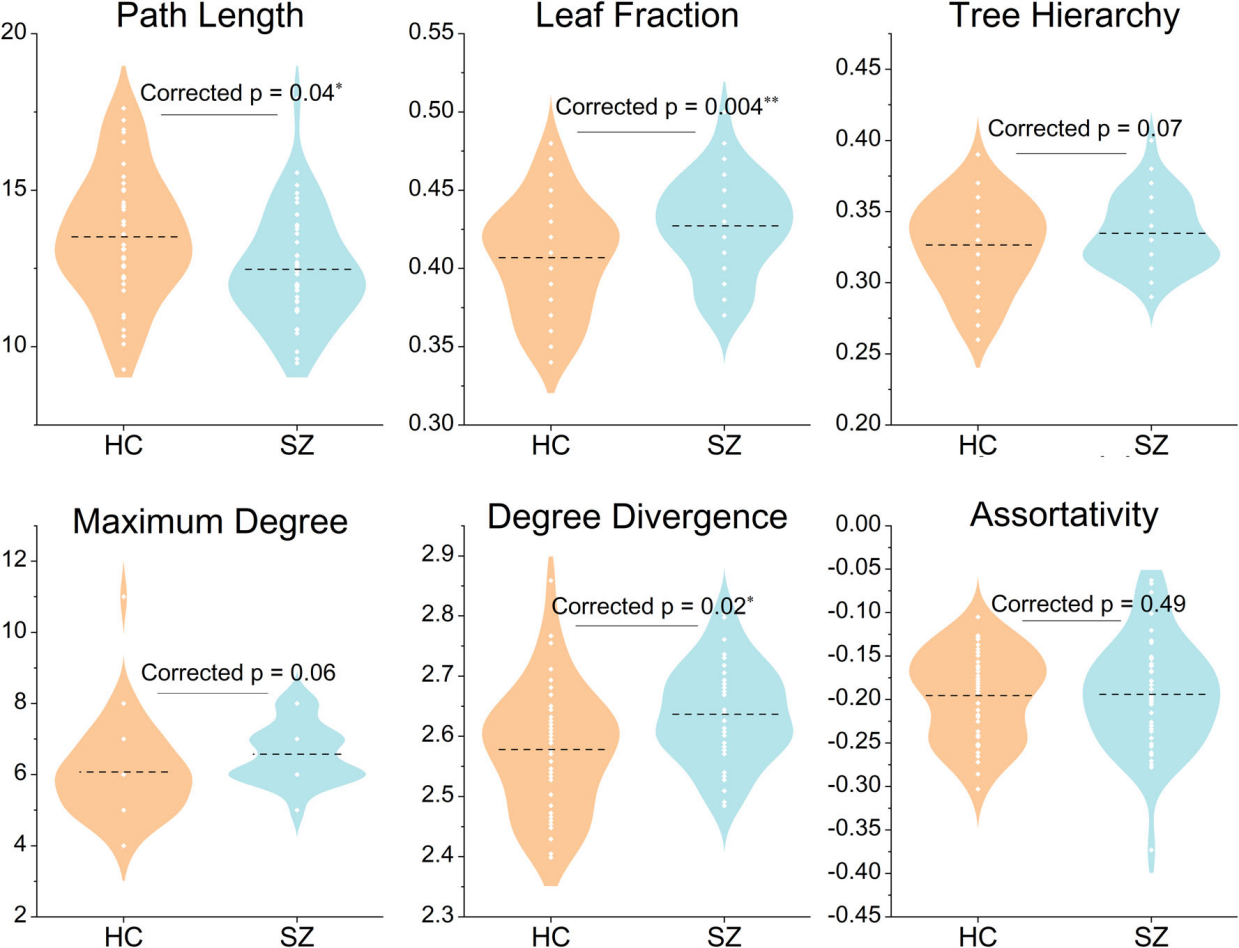
Between‐group differences of tree metrics. HC, healthy control, SZ, schizophrenia; “*” indicates significant difference (corrected *p* <.05 for 5,000 permutations), ** for *p* <.01

The group‐level difference in MST topology was further quantified by comparisons between multiple global MST metrics, and the results are shown in Figure 5. We observed significantly reduced path length (FDR corrected *p* = .04) in patients, indicating greater global network integration. Increased leaf fraction (FDR corrected *p* = .004) and increased degree divergence (FDR corrected *p* = .02) in patients further revealed a more centralized network configuration with the presence of more low‐degree peripherals, and thus more nodes with prominently high degree. These results convergently delineated a more integrated, star‐like MST configuration in schizophrenia, suggesting the breakdown of the optimal balance between integration and segregation.

In our validation analysis, we found significant difference between MST structure of patients and healthy controls revealed by similarity test under two of three additional parcellation schemes. In addition, an identical pattern of network aberration manifested itself for all three parcellations through quantitative analysis, characterized by higher leaf fraction, shorter path length, and greater degree divergence. The result was also generally unchanged when using wavelet despiking for additional head motion control, removing four time points, and under global signal regression (details are shown in Supporting Information). Taken together, these findings highlighted the deviation from the optimal MST structure in patients toward a more centralized network accompanied with enhanced integration and decreased segregation.

### 3.3 Regional network organization and group‐level MST degree distribution

We compared nodal level connector index and degree by permutation tests, and then identify prominent hubs and connectors in the group‐averaged MST. Although no significant difference was observed in nodal degree centrality, we found significantly reduced connector index (corrected *p* = .03) of dorsal frontal cortex (MNI coordinate: [−42 7 36]) in schizophrenia patients, indicating a loss of ability to connect different brain regions. However, the reduced ci did not correlate with the five behavior dimensions we studied.

**FIGURE 6:**
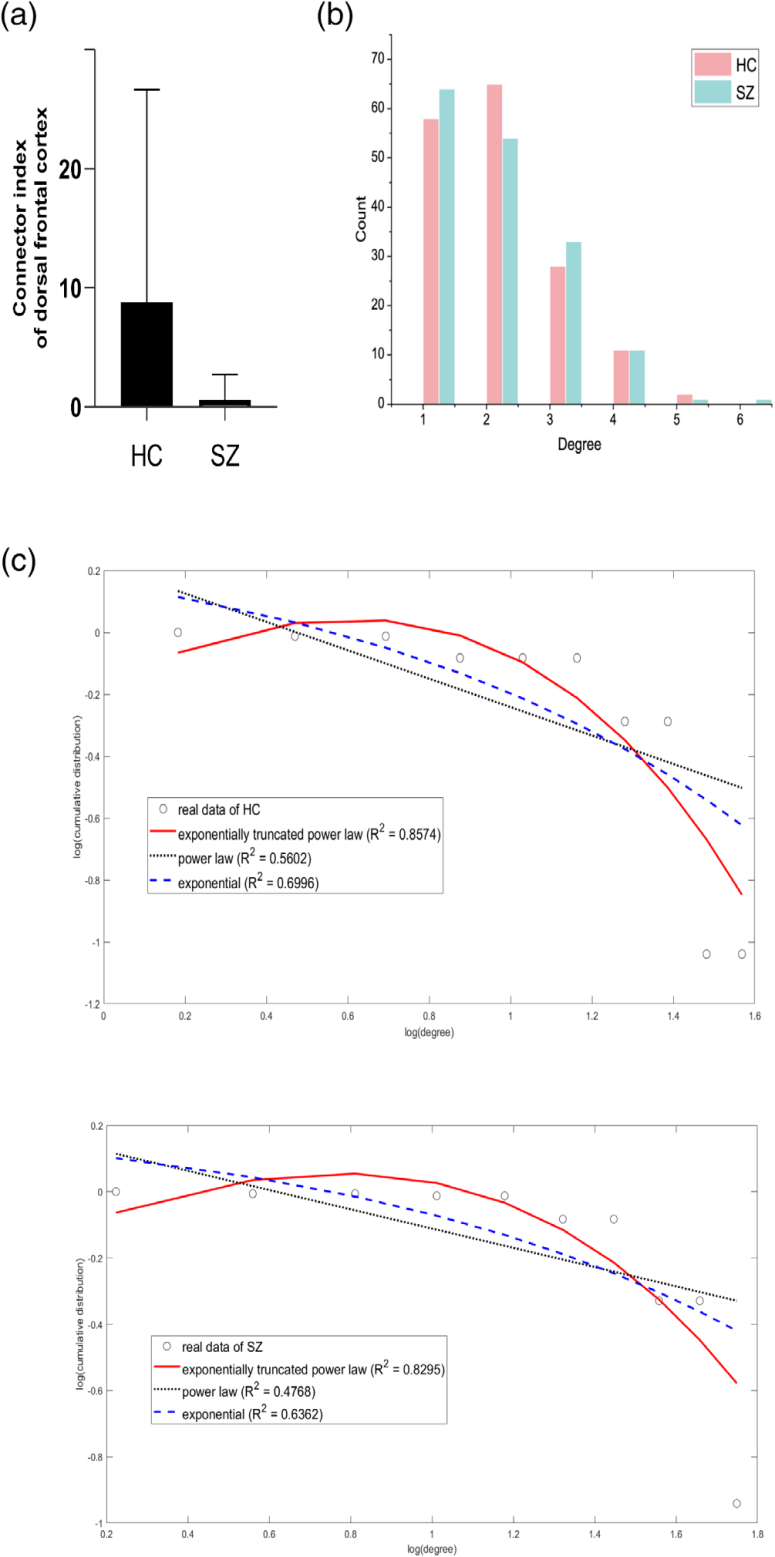
(a) Significantly reduced connector index in dorsal frontal cortex (corrected *p* = .03). (b) The degree distribution of group‐level MSTs. (c) Fitting plot of the two distribution to exponential distribution, power law distribution and exponential truncated power law distribution. HC, healthy control; SZ, schizophrenia. The fitting and graph were completed using GRETNA toolbox (J. Wang, Wang, Xia, Liao, & Evans, 2015)

A demonstration of the degree distribution of the two group‐averaged MSTs is shown in Figure 6. As expected, higher number of low‐degree nodes were observed in schizophrenic group, consistent with the findings obtained by hypothesis tests. We found that exponentially truncated power law distribution was the best fit for group‐averaged MSTs of both the healthy group and the schizophrenia group (Figure 6), in line with a previous study (Achard et al., 2006). In particular, the *R*‐squared value for healthy group was 0.8574 for exponentially truncated power law distribution, 0.5602 for power‐law distribution, and 0.6996 for exponential distribution (values closer to 1 indicate good fit). In schizophrenia group, *R*‐squared values were 0.8295, 0.4768, and 0.6392 for the three distributions, respectively. In addition, the parameters for exponentially truncated power law distribution were *α* = 2.3700 and *k*_*c*_ = 1.3427 in healthy group, and *α* = 1.3060 and *k*_*c*_ = 2.3195 in schizophrenia group. The better fit of two distributions to the exponentially truncated power law indicated that the brain networks tended to scale‐free network compared to a random network.

**FIGURE 7:**
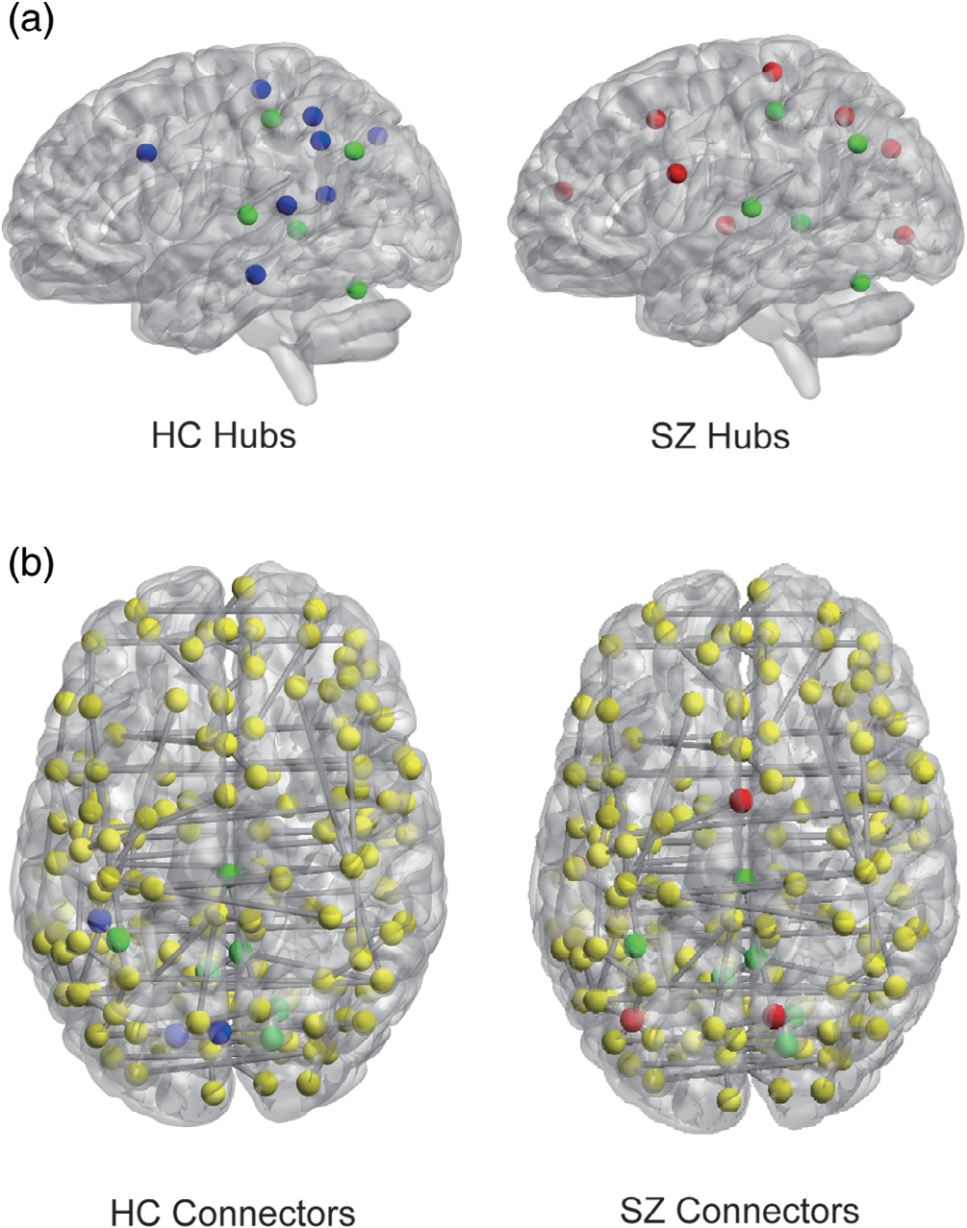
Locations of network hubs and connectors in the brain. Nodes in green are hubs common to both groups; nodes in red are hubs/connectors specific to schizophrenia group; nodes in blue are hubs/connectors specific to healthy control group. Nodes in yellow are all remaining nodes that are not hubs nor connectors. HC, healthy control; SZ, schizophrenia. The graphs were generated using BrainNet Viewer toolbox (Xia, Wang, & He, 2013)

In addition, we recorded nodes with degree or connector index 2 *SD* higher than average values in the group‐level MST as hubs or connectors respectively. The remaining nodes could be regarded as less important peripheral nodes (Figure 7). There was no overlap between hubs and connectors in both MSTs, indicating successful differentiation between the two kinds of roles. Locations and names of hubs and connectors for both groups are listed in Table 3.

**TABLE 3:** Locations of hubs and connectors identified in the group‐level MST and the intrinsic networks they belong to

	MNI coordinate			Network
Group and region	*X*	*Y*	*Z*	
Hubs‐HC				
medial Cerebellum	−16	−64	−21	Cerebellum
Basal ganglia	−6	17	34	Cingulo‐opercular
angular Gyrus	−48	−63	35	Default
inferior Temporal	−59	−25	−15	Default
Occipital	−9	−72	41	Default
posterior Cingulate	−8	−41	3	Default
posterior Cingulate	−5	−52	17	Default
IPL	−48	−47	49	Fronto‐parietal
IPL	−53	−50	39	Fronto‐parietal
Parietal	−38	−27	60	Sensorimotor
posterior Parietal	−41	−31	48	Sensorimotor
Temporal	−53	−37	13	Sensorimotor
Temporal	−54	−22	9	Sensorimotor
Hubs‐SZ				
medial Cerebellum	−16	−64	−21	Cerebellum
medial Frontal Cortex	0	15	45	Cingulo‐opercular
middle Insula	32	−12	2	Cingulo‐opercular
angular Gyrus	−48	−63	35	Default
posterior Cingulate	−8	−41	3	Default
ventromedial Prefrontal Cortex	9	51	16	Default
IPS	−32	−58	46	Fronto‐parietal
Occipital	−16	−76	33	Occipital
posterior Occipital	33	−81	−2	Occipital
Parietal	−24	−30	64	Sensorimotor
posterior Parietal	−41	−31	48	Sensorimotor
Temporal	−54	−22	9	Sensorimotor
ventral Frontal Cortex	−55	7	23	Sensorimotor
Connectors‐HC				
Occipital	−2	−75	32	Default
posterior Cingulate	1	−26	31	Default
Precuneus	5	−50	33	Default
Precuneus	−6	−56	29	Default
IPL	−41	−40	42	Fronto‐parietal
posterior Parietal	−35	−46	48	Fronto‐parietal
Occipital	17	−68	20	Occipital
Occipital	−16	−76	33	Occipital
Occipital	15	−77	32	Occipital
Parietal	−55	−22	38	Sensorimotor
Connectors‐SZ				
IPS	−36	−69	40	Default
posterior Cingulate	1	−26	31	Default
Precuneus	5	−50	33	Default
Precuneus	11	−68	42	Default
Precuneus	−6	−56	29	Default
posterior Parietal	−35	−46	48	Fronto‐parietal
Occipital	17	−68	20	Occipital
Occipital	15	−77	32	Occipital
precentral Gyrus	−54	−22	22	Sensorimotor
SMA	0	−1	52	Sensorimotor

*Note*: Regions are ordered alphabetically based on networks they belong to.

Abbreviations: HC, healthy control; SZ, schizophrenia.

Generally speaking, compared to healthy subjects, group‐level MST from schizophrenic patients exhibited a shift in hub locations with more hubs emerging in frontal and occipital regions, in line with previous studies (A. F. Alexander‐Bloch et al., 2013; L. Wang et al., 2010). Specifically, medial frontal cortex, ventromedial prefrontal cortex, ventral frontal cortex, and two occipital regions emerged as new hubs in schizophrenia group. The functions of these regions could be understood using the notion of intrinsic functional networks. Adopting the notion of Dosenbach's network partition scheme, we found that hubs of both groups were widely distributed in cingulo‐opercular, default, fronto‐parietal, and sensorimotor networks (Dosenbach et al., 2010).

Meanwhile, we found that major connectors in both groups were primarily located in occipital and precuneus, and 60% of main connectors were shared between groups, suggesting that connectors generally did not change location in the disorder. We also found that a large portion of connectors emerged in the default mode network (Table 3). It can thus be inferred that the default mode network may serve as an important transfer station for neural information communication.

### 3.4 Structural equation modeling

We used SEM to test indirect effect of network integration with cognitive performance as outcomes in each group and in mixed populations, also with clinical symptoms as outcomes for schizophrenic group.

We found that among other models, the one with clinical symptoms as outcome variables reached satisfactory model fit (PPP = .155). As expected, age was positively correlated with leaf fraction in patients (95% CI [0.075, 0.740]), indicating greater level of network integration with age. Notably, this relation was also valid when tested for both groups mixed (*p* = .008, standardized beta value = .295). We also found that severity of negative symptoms was positively correlated with leaf fraction (95% CI [0.194, 0.817]). Specifically, we identified a significant indirect effect of age on negative symptom through leaf fraction (95% CI [0.026, 0.449]). Primary parameters of the model were summarized in Figure 3b. Similar results were obtained when using a latent variable “network structure” estimated from six graph metrics instead of using leaf fraction solely (Supporting Information). The result confirmed our hypothesis that aging would lead to reduced functional segregation, which then induces greater symptom severity. With additional head motion control and removal of four time points, the effect was again found to remain significant, but the effect was nonsignificant when using global signal regression (Supporting Information). Other mediation models with cognitive outcomes either fail to reach satisfactory model fit or the mediation effect was not significant (Supporting Information). Also, there was no significant correlation found between the brain network metric and cognitive test scores. In our moderation analysis, we found a nonsignificant moderation effect (*p* = .95), suggesting that the influence of age on leaf fraction was not affected by the disease (Supporting Information).

## 4 DISCUSSION

In the present study, we evaluated functional brain network changes in schizophrenia with MST representation and its relation to behavior, combining graph theoretical analysis and SEM. We demonstrated that the regular interaction pattern between functional integration and segregation was disrupted in schizophrenia and the dysregulation was related to both age and behavioral outcomes. The SEM analysis further suggested a potential mediation mechanism for these variables. In addition, we defined a connectivity‐transitivity framework to analyze nodal properties in MSTs and found reduced transitivity in the dorsal frontal cortex. Based on the two‐dimensional approach, we also revealed reconfiguration patterns of the spatial distribution of major brain connectors and hubs in schizophrenia patients. Our MST‐based work shed new light on the aberrant functional brain network and age–brain–behavior interaction in schizophrenia.

### 4.1 MST as a promising framework for unbiased cross‐disorder network comparison

The present study used MST as an unbiased network representation method, which dispenses the need of selection of binary or weighted network, different threshold values and normalization techniques. In addition, it has been shown that MST is robust against random noise (Otte et al., 2015), and insensitive to variations in connection densities and average connection strength (Otte et al., 2015; Stam et al., 2014; Tewarie et al., 2015). This is particularly useful for the analysis of brain network in different categories of populations, such as different age groups (Boersma et al., 2013; He et al., 2019; Smit et al., 2016) or populations of distinct clinical status as did in the present study, because MST eliminates the confounds caused by heterogeneous connection densities, enabling us to focus on the critical skeleton of brain networks. Importantly, using both simulation and empirical data, analysis of MST metrics has been demonstrated to be able to reveal meaningful topological properties of the original brain network (Tewarie et al., 2015; van Dellen et al., 2018).

Implications of the irregular MST organization observed in patients with schizophrenia could be elaborated adopting the recently proposed “MST network space” model, which can be seen as the MST counterpart of the small‐world network space (He et al., 2019). Through studying developmental trajectories of MST during childhood, He and colleagues noted that, optimal MST structures emerged during brain maturity seeking a balance between integration and segregation. For MSTs, integrated organization resembles a star‐like structure, characterized by a high leaf fraction, a shorter path length, and a greater degree divergence; on the other hand, a line‐like MST symbolizes extreme segregation (Figure 2). Our findings of excessively integrated MSTs in schizophrenia could be seen as a deviation from the optimal configuration. Such redundant investment in neural resources to bring greater global integration was noted as a significant signature on the spectrum of brain disorders (van den Heuvel & Sporns, 2019). Projecting different pathological trajectories onto the MST network space, disruptions found in other psychosis diseases could also be understood as a shift from normal state: compared to healthy controls, a move toward more star‐like configuration (lower‐right corner in Figure 2) were observed in Attention‐deficit/hyperactivity disorder (ADHD) (Janssen et al., 2017) and major depressive disorder (X. Li et al., 2017); on the contrary, a move toward line‐like MST organization was found in Multiple Sclerosis (Tewarie et al., 2014), delirium (van Montfort et al., 2018), and behavioral variant Frontotemporal Dementia (bvFTD) (Saba et al., 2019). Crucially, since the methodological heterogeneity is minimized when MST is adopted, direct comparisons of network topologies across disorders can be plausible. A cross‐disorder landscape of MST brain networks can be depicted with individual disorders positioned based on the relative locations of their MSTs in the MST network space. (He et al., 2019; M. Yu et al., 2016).

### 4.2 A significantly more integrated network structure in schizophrenia and its interactions with age and clinical symptoms

Our analysis based on MST metrics revealed an excessively integrated, star‐like brain network in patients with schizophrenia. As the critical backbone of the original network, MST structure could reflect fundamental topological alterations in the brain network in schizophrenia. It is worth noting that despite a significant inconsistency in graph theoretical studies of schizophrenia (Fornito, Zalesky, Pantelis, & Bullmore, 2012; van den Heuvel & Fornito, 2014), our findings of a more centralized MST in schizophrenia were validated under three other parcellation schemes and resonate with two previous schizophrenia pathology studies based on resting‐state EEG paradigm (Jonak et al., 2019; Krukow et al., 2019). In the first study, higher leaf fraction and reduced network diameter in MSTs in several frequency bands was found in schizophrenia patients; in the second study, patients with longer illness duration were found to exhibit more star‐like MST, characterized by higher leaf fraction and tree hierarchy index (closer to 0.5). Given significant methodological heterogeneities in classical network analysis, the convergent findings of MST studies may be a more convincing corroboration of a more centralized functional network in schizophrenic patients. This pattern was also noted in some traditional graph theoretical studies (A. F. Alexander‐Bloch et al., 2010; A. F. Alexander‐Bloch et al., 2013; Lynall et al., 2010), where increased global efficiency was reported.

To understand the effect of intensified network integration, a heuristic elaboration from a graph theoretical perspective could be inspired by associating with the cascading network failure hypothesis (Jones et al., 2016; van den Heuvel & Sporns, 2019). Analogous to cascade failures in power gird networks, over‐centralized configuration observed in schizophrenia MSTs could be a result of the redistribution of the workload of other dysfunction nodes. In other words, the load of some initially failed nodes may be erroneously allocated to adjacent hubs. These functional hubs may then be overloaded, connecting to too many “leaves,” thus the creation of rich‐club is prevented (van den Heuvel & Sporns, 2011) and their own functioning may also be damaged. Similarly, the presence of more leaves and possibly overloaded hubs has been noted and quantitatively elaborated in our study. As a result, overall network performance would possibly be jeopardized, leading to degeneration of clinical and cognitive outcomes in the disorder. The cascade failure theory has been used to explain network dysconnectivity in Alzheimer's disease spectrum (Jones et al., 2017, 2016). Other alternative explanations of the negative effect of enhanced integration posit that the increase of densely connected hubs would render the brain more vulnerable to brain disorders, since the dysfunction of hubs would result in greater influence on the network performance (Crossley et al., 2014; van den Heuvel & Sporns, 2013). Our finding of a positive association between enhanced integration and schizophrenia negative symptoms provides novel evidence of the negative influence of excessively centralized network configuration.On the other hand, increased leaf fraction, degree divergence and decreased path length found in our study can also be construed as a sign of impaired functional segregation. Obviously, with more nodes collectively connected to a few prominent hubs, it would be difficult to form specialized neural systems. One could imagine that in the extreme situation where all nodes in the network except one were connected to a hub, there is no functional specialization at all because there is only one “processing unit.” The phenomenon was also noted in aging‐related brain network topological studies where decreased functional segregation throughout adult lifespan were consistently reported (Cao et al., 2014; Chan, Park, Savalia, Petersen, & Wig, 2014; Ferreira et al., 2016; Geerligs et al., 2015), and the failure of achieving high‐level functional specialization had been shown to undermine cognitive performance and long‐term memory (Chan et al., 2014; Ferreira et al., 2016). In line with previous research, we also found a positive correlation between subject age and leaf fraction through adulthood (both for healthy group and patients), suggesting that aging is accompanied by the loss of segregation. Importantly, under the SEM framework, we successfully interpreted the complex interactions between age, integration and clinical symptom severity in schizophrenia, suggesting an indirect interaction mechanism in which increased age lead to greater level of network integration, which then induce aggravated negative symptoms. The influence of age on network changes was also shown to be unaffected by the disease in our moderation model.

Previous studies often indicate that clinical symptoms assessed by clinical tests were either negatively or not significantly correlated with age (Friedman, Harvey, Kemether, Byne, & Davis, 1999; Schultz et al., 1997). Other researchers also pointed out that this may be due to cohort effect and survivor bias (Jeste, Wolkowitz, & Palmer, 2011). Indeed, the (total) correlation effect between age and symptom severity was found to be nonsignificant in our study. However, with the aid of mediation analysis, we found that the influence of age may be a more complex, indirect effect through a third variable, namely network structure, instead of a direct one. (It should be noted that a significant “total” effect between independent and dependent variables is not a “gatekeeper” for analyzing indirect effect of a third variable [Hayes, 2009]). This might provide the reason for aggravated symptoms in schizophrenia with time if clinical treatment is not presented. In other words, the mediation effect may indicate that the transition of brain network toward integrated structure in the need of support cross‐domain information fusion and processing, as a natural process during aging, would also result in higher level of schizophrenia symptom severity. Interestingly, this interpretation coincides with a finding from genetic studies that schizophrenia may be an undesired byproduct of the human brain's capabilities through the evolution of greater cognitive abilities (Khaitovich et al., 2008; Scarr, Udawela, & Dean, 2018).

### 4.3 Regional level reconfiguration patterns in schizophrenia revealed by connectivity‐transitivity framework

Concerning regional level analysis, we proposed a two‐dimensional approach suitable for MST to classify nodes into hubs, connectors and peripherals based on their connectivity and transitivity. A similar framework was devised for rich‐club analysis, in which links in the brain network were categorized into feeder connections, rich club connections, and local connections based on what kind of nodes (hubs or peripherals) they were connecting (Collin, Kahn, De Reus, Cahn, & Van Den Heuvel, 2014; van den Heuvel et al., 2013; Van Den Heuvel, Kahn, Goñi, & Sporns, 2012). However, this classification scheme only considered connectivity and ignored transportation properties. In addition to the framework based on the notion of rich club, some researches evaluated nodal roles in brain network according to their within‐module and between‐module connectivity (Guimerà, 2005) and nodes with high participation coefficient was thought to be crucial to facilitate intermodule communication (Rubinov & Sporns, 2010). This framework is also nonapplicable to MST because of the absence of modular structures. Compared to existing methods, our proposed approach exploited the simplicity of MST structure and investigated transportation properties, which are often ignored in previous MST research. Under this two‐dimensional framework, a more complete landscape of nodal characteristics in MST could be depicted.

In this study, we found that in healthy control group hubs were mostly distributed in the parietal and posterior temporal regions, which is consistent with previous traditional graph theoretical studies (A. F. Alexander‐Bloch et al., 2013; Rubinov & Bullmore, 2013) in which nodes with high centrality were found to be in these regions. Using MST, we defined graph metrics for connectors and found that connectors were largely over occipital and posterior regions. Our proposed “connector index” highlighted the differentiation between “hubs” and “bridges,” thus connectors were identified in this study based on their ability of facilitate the brain's global communication, while previous studies mostly focus on centrality. These results indicate that occipital and posterior regions may be crucial in information transfer across the brain. Analysis of group‐level averaged MST revealed a shift of hub location with more hubs emerging in frontal regions (e.g., ventromedial prefrontal cortex, medial frontal cortex) in schizophrenia, an aberration also noticed by some classical brain network studies (A. F. Alexander‐Bloch et al., 2013; L. Wang et al., 2010). This phenomenon might be linked to the “overloading” hypothesis as discussed above.

Compared to observed location shift of hubs, we found that the spatial locations of connectors were largely retained in schizophrenia, indicating that most major connectors in the brain network remained robust against the disease. However, we found that the connector index of dorsal frontal cortex was significantly reduced in patients with schizophrenia. The dysconnectivity of dorsal and frontal regions have been noticed in enormous studies of schizophrenia (Minzenberg, Laird, Thelen, Carter, & Glahn, 2010). As a complement, our study provided a new perspective, suggesting that the function of these regions to facilitate global communication might be damaged in the disease. However, the abnormal pattern did not correlate with the five behavior dimensions we studied. Further research may be needed to explore the cognitive implications of the loss of transitivity in brain network. We also found that a large portion of group‐level connectors belonged to the default mode network, suggesting that the network may be responsible for information transfer during resting state.

### 4.4 Limitations

There are several limitations in this study. First, the sample size of 80 may be considered insufficient for structural equation model analysis. To address this problem, we employed Bayesian methods for parameter estimation, which was recommended for relatively moderate sample size. Nonetheless, the statistical inferences need to be validated in a larger population. Second, the findings of the present study were purely driven by rs‐fMRI data and statistical analysis, which may be further examined by metabolic evidence and multimodal neuroimaging techniques. Third, albeit successful interpretation of our model, other phenotypic variables, such as gender and handedness (Biswal et al., 2010; M. Li et al., 2014), may also influence network structure and behavior outcomes of both patients and controls. Specifically, to the best of our knowledge, the current dataset did not include statistics of age at onset and illness duration of patients. Since these two factors are also correlated with age, it would be valuable to explore the roles of these variables in our SEM. Thus, our model may not be a complete portrait of complex interactions among these variables. Future studies could approach the problem by incorporating more potential factors and conduct analysis in a more extensive population. In addition, despite its simplicity and unbiased nature, the MST method has certain limitations since it requires an all‐positive network as input and forbids any loops. The interpretation strategy of negative connections has been contentious in brain network studies (Schwarz & McGonigle, 2011), and it is difficult to study modular structures in MST due to the absence of loops. In this study, we simply defined hubs as independent functional units, but an alternative hierarchical clustering view has been proposed on MSTs (M. Yu et al., 2015). Further studies are needed to address MST's ignorance of negative connections and explore its potential modular structure.

## 5 CONCLUSION

In conclusion, the current study demonstrated disrupted MST structure in schizophrenia patients, characterized by excessive integration. The behavioral relevance of the aberration was further illustrated by SEM‐based mediation analysis, suggesting that aging may exert indirect positive influence on schizophrenia negative symptom severity through MST structure of brain network. Taken together, these results may improve our understanding of intertwined interaction patterns among multiple behavioral and connectomic variables in neuroimaging studies of schizophrenia. In addition, we found significantly reduced transitivity in dorsal frontal cortex in schizophrenia. We also revealed a shift of hub locations and largely unchanged connector locations, providing new insights into reconfiguration patterns of regional network structures in the disorder.

## Supporting information


**Appendix** **S1:** Supporting Information

## Data Availability

All the data used is from open access data. The dataset used in this study is from publicly available OpenNeuro repository with accession number ds000030 (https://openneuro.org/datasets/ds000030/versions/00016).
