# Supplementary material for: Disentangling age‐ and disease‐related alterations in schizophrenia brain network using structural equation modeling: A graph theoretical study based on minimum spanning tree
Source: Hum Brain Mapp. 2021 May 7;42(10):3023–41. doi: 10.1002/hbm.25403 (PMC8193510; doi:10.1002/hbm.25403)
Supplement: Supplementary file 1 — Appendix S1: Supporting Information [file HBM-42-3023-s001.pdf]

Supplementary Material

1. Methods and Results

1.1 Medication use for schizophrenia patients

35 of 40 schizophrenia patients were exposed to antipsychotic medication at the time of scanning. We included medication usage as a covariate in our structural equation model by specifying it as an exogenous predictor of mediators and outcome variables. To do so, we converted antipsychotic medication dosage to its chlorpromazine-equivalent value by using ratios presented in (Kroken, Johnsen, Ruud, Wentzel-Larsen, & Jørgensen, 2009; Leucht et al., 2014). Antipsychotics were not further classified into typical (first generation) and atypical (second generation) since only few patients were using typical antipsychotics. The details of medication involved in our sample were summarized in **Table S1** (adapted from (Kebets et al., 2019)).

1.2 Correlation between variables

Coefficients and p values of inter-correlations among the six tree metrics (path length, leaf fraction, tree hierarchy index, maximum degree, assortativity, degree divergence) were summarized in **Table S2**. In general, these variables were correlated with each other, since the network structural implications they reflected are convergent. For example, increased leaf fraction and decreased path length both indicate strengthened network integration, thus they are negatively correlated.

Partial correlation between predictors (age, tree metrics) and outcomes (the five behavior variables) were shown in **Table S3**. The partial correlations were generally small and not significant, and coefficients were small, indicating that no single variable can predict cognitive or clinical outcomes. Thus, there may me more complex interactions among them.

1.3 Structural equation model parameters

We replicate here details of four structural equation models we tested:

Model 1: Mediation model: (age)- (leaf fraction)- (working memory, executive function). The sample only includes 40 healthy individuals.
Model 2: Mediation model: (age)- (leaf fraction)- (working memory, executive function). The sample includes 40 healthy subjects and 40 schizophrenia patients.
Model 3: Mediation model: (age)- (leaf fraction)- (psychotic symptom, negative symptom, disorganization).
The sample includes 40 schizophrenia patients. The three latent variables were based on previously established three-factor structure (Andreasen, Arndt, Alliger, Miller, & Flaum, 1995).
Model 4: Moderation model: The influence of age on leaf fraction were moderated by schizophrenia (as a categorical variable). The sample includes all 80 subjects.
The first three models' parameters were summarized in **Table S4, S5.** In model 4, we found a non-significant moderation effect (p = 0.85).

We report the estimate of parameter posterior distribution and the 95% credible interval (CI) in brackets. A CI with 0 falling outside indicates significant effect.

1.4 Validation analysis

1.4.1 Replication under different atlases

We replicate our similarity and global level comparisons in three addition parcellation scheme: (1) Harvard-Oxford 112-ROI atlas (Tzourio-Mazoyer et al., 2002); (2) Power's 264-ROI atlas (Power et al., 2011); (3) Schaefer's 400-ROI atlas (Schaefer et al., 2018). We found that there was significant MST structural difference revealed by similarity test in two of three parcellations (p = 0.08 for Harvard-Oxford 112-ROI atlas, p = 0.035 for Power's 264-ROI atlas, p = 0.018 for Schaefer's 400-ROI atlas). Moreover, a similar pattern of MST alterations in all three parcellations was found through comparisons between tree metrics (**Figure S2, S3, S4**). The between-group comparison outcomes for the original Dosenbach-atlas was also similar to the result presented in the main text without additional head motion control (**Figure S5**)

1.4.2 Replication with additional head motion control

To further reduce motion-related artifacts, we applied wavelet despiking to BOLD time series to remove spurious signal fluctuations resulting from non-neuronal confounds. In this method, a maximum-overlap discrete wavelet decomposition (MODWT) was first applied to the time courses of each voxel. Outlying wavelet coefficients at different frequency scales were regarded as artifacts and then removed, thus only "signal" coefficients were retained for the subsequent inverse MODWT to construct time series for further analysis.

As a result, we found that the between-group comparison outcomes for the original Dosenbach-atlas was also similar to the result presented in the main text without additional head motion control (**Figure S5**). In addition, we fitted the Dosenbach data with wavelet despiking again using the structural equation model. As expected, we found a significant mediation effect (Standardized effect size 0.164, 95% CI [0.016 0.420] among age, leaf fraction and negative symptom severity.

1.4.3 Replication with different variables for SEM

The associations with cognitive functions were not significant in either partial correlation or BSEM analyses. In the meantime, factor loadings of CPT-D Prime and Stroop task on the executive function were not significant. It can be possible that the executive function latent variable estimation was biased towards cognitive processing measured by verbal fluency task specifically. To test this, we performed a further exploratory mediation analysis where verbal fluency score acted as the mediator between age and network structure. The result indicates that the mediation effect is not significant (95% CI [-0.161 0.014]) and there is no significant correlation between either network structure and verbal fluency score (95% CI [-0.410 0.062]) nor age and verbal fluency score (95% CI [-0.144 0.328]). Thus, we observe no significant effect even using verbal fluence score alone as the outcome.

In addition, we performed another validation analysis in which a latent variable called “network structure” was extracted from six graph metrics, and then used for mediation analysis in SEM. The resulted model details are shown in Table S6. Four of six network metrics showed significant factor loadings, and the indirect effect exerted by network structure on the influence between age and negative symptom severity was significant (95% CI [0.010 0.422]).

1.4.4 Replication using removal of more time points

To further improve the robustness of our results, we conducted an additional analysis in which four time points, instead of two, were removed as the first step of pre-processing to protect the stable quality of imaging.

The results for comparisons of tree metrics are shown in **Figure S6**. Generally, the results are consistent with what was reported in the main text, indicating a higher level of network integration in schizophrenia patients. In addition, we again found a significant mediation effect of leaf fraction on age and negative symptom severity (95% CI [0.016 0.605]).

1.4.5 Replication using global signal regression

Global signal has always been a contended topic and there are many pros and cons about removing it. In this study, we recognize that processing strategy of global signals does have an impact on our findings due to the nature of MST algorithm. The algorithm requires an all-positive network as input and output a connected subgraph while seeking to retain strongest connections. Namely, contrast to traditional studies, only a fraction of positive edges is used in the algorithm. In the meantime, it has been reported that removal of global signal would result in a functional connectivity distribution in which approximately half of edges are negative (Murphy, Birn, Handwerker, Jones, & Bandettini, 2009). This alteration in distribution shape would clearly result in a different MST for the original network because the algorithm uses only the strongest connections (while ensuring that the graph is connected). To sum up, applying GSR would possibly change the sign of some of connections in the network, thus result in an altered MST.

In addition, previous studies has shown that global signal may contain subject-specific and disease-related information that should not be ignored especially in pathological studies (Chen et al., 2018). Specific to schizophrenia, it has been found that global signals may contain neurobiologically meaningful information about the schizophrenia disease and can improve diagnostic specificity (Yang et al., 2014). Thus, we chose not to remove global signal in the main text to prevent loss of potentially important information.

We performed a replication study where GS was removed to evaluate the effect of this step. The results of between-group comparisons are summarized in Figure S7. We found that all graph metrics except for characteristic path length showed non-significant between-group difference. The characteristic path length of healthy controls was found to be significantly higher than that of patients (corrected p

= 0.04), indicating the robustness of our results to a certain degree. However, we failed to identify a significant mediation relationship among age, leaf fraction and negative symptom severity (95% CI [-0.136, 0.164]).

Tables

Table S1. Antipsychotic medication involved in the sample.

Medication / Molecule	Targeted neurotransmitter system(s)
Abilify / Aripiprazole	Serotonin, Dopamine
Clozaril / Clozapine	Serotonin, Norepinephrine, Dopamine
Fanapt / Iloperidone	Serotonin, Norepinephrine, Dopamine
Geodon / Ziprasidone	Serotonin, Dopamine
Haldol / Halope	Dopamine
Invega / Paliperidone	Serotonin, Norepinephrine, Dopamine
Loxitane / Loxapine	Serotonin, Dopamine
Prolixin / Fluphenazine	Dopamine
Risperdal / Risperidone	Serotonin, Norepinephrine, Dopamine
Saphris / Asenapine	Serotonin, Norepinephrine, Dopamine
Seroquel / Quetiapine	Serotonin, Dopamine
Zyprexa / Olanzapine	Serotonin, Dopamine

Table S2. Intercorrelation between tree metrics. *: p<0.05; **: p<0.01.

		L	lf	Th	D_{max}	κ	r
L	Pearson Correlation Coefficient	1	-.571**	-.150	-.421**	-.572**	-.383**
	Two-tailed p-value		.000	.183	.000	.000	.000
	Sample size	80	80	80	80	80	80
lf	Pearson Correlation Coefficient	-.571**	1	.645**	.483**	.887**	-.129
	Two-tailed p-value	.000		.000	.000	.000	.253
	Sample size	80	80	80	80	80	80
Th	Pearson Correlation	-.150	.645**	1	.289**	.562**	-.160

	Coefficient						
	Two-tailed p-value	.183	.000		.009	.000	.157
	Sample size	80	80	80	80	80	80
D_{max}	Pearson Correlation Coefficient	-.421**	.483**	.289**	1	.677**	.192
	Two-tailed p-value	.000	.000	.009		.000	.088
	Sample size	80	80	80	80	80	80
κ	Pearson Correlation Coefficient	-.572**	.887**	.562**	.677**	1	-.049
	Two-tailed p-value	.000	.000	.000	.000		.665
	Sample size	80	80	80	80	80	80
r	Pearson Correlation Coefficient	-.383**	-.129	-.160	.192	-.049	1
	Two-tailed p-value	.000	.253	.157	.088	.665	
	Sample size	80	80	80	80	80	80

Table S3. Partial correlations between predictor variables and outcomes.

Predictors	Partial Correlation Coefficient / p-value				
	Executive Function	Working Memory	Negative Symptom	Paranoia	Disorganization
Age	0.105 / 0.556	-0.010 / 0.957	0.021 / 0.908	0.028 / 0.876	0.074 / 0.678
L	0.122 / 0.491	0.056 / 0.752	0.091 / 0.608	-0.320 / 0.065	-0.043 / 0.810
lf	0.221 / 0.208	0.274 / 0.117	0.233 / 0.185	0.049 / 0.782	0.049 / 0.782
Th	-0.041 / 0.818	-0.213 / 0.227	0.070 / 0.693	0.047 / 0.791	0.014 / 0.936
D_{max}	0.255 / 0.145	0.198 / 0.263	-0.056 / 0.752	-0.405 / 0.801	0.056 / 0.753
κ	-0.216 / 0.220	-0.227 / 0.196	0.255 / 0.146	-0.189 / 0.284	-0.049 0.783
r	-0.039 / 0.825	0.107 / 0.548	0.239 / 0.174	0.000 0.998	-0.118 0.505

Table S4. Model parameters for model 1 and 2.

	Model 1 (n = 40) parameter [95% CI] (PPP = 0.068)	Model 2 (n = 80) parameter [95% CI]
Structural Model Pathways		
Age - Leaf Fraction	**0.337 [0.011, 0.669]**	**0.359 [0.126, 0.583]**
Leaf Fraction – Working Memory	-0.126 [-0.480, 0.232]	-0.204 [-0.435, 0.012]
Leaf Fraction – Executive Function	0.016 [-0.385, 0.436]	-0.212 [-0.444, 0.010]
Age – Working Memory	0.206 [-0.140, 0.548]	0.060 [-0.168, 0.286]
Age – Executive Function	0.178 [-0.212, 0.573]	0.204 [-0.022, 0.436]
Indirect Effects		
Age – Leaf Fraction – Working Memory	-0.032 [-0.200, 0.082]	-0.068 [-0.186, 0.003]
Age – Leaf Fraction – Executive Function	0.003 [-0.153, 0.175]	-0.071 [0.190, 0.003]
Control Pathways		
Gender – Working Memory	-0.067 [-0.811, 0.653]	-0.069 [-0.536, 0.401]
Framewise Displacement (Rotation) – Working Memory	-0.580 [-1.275, 0.060]	-0.243 [-0.570, 0.067]
Framewise Displacement (Translation) – Working Memory	0.137 [-0.451, 0.779]	-0.165 [-0.488, 0.155]
Gender – Executive Function	-0.179 [-0.991, 0.628]	-0.251 [-0.723, 0.203]
Framewise Displacement (Rotation) – Executive Function	-0.195 [-0.996, 0.562]	0.009 [-0.310, 0.327]
Framewise Displacement (Translation) – Executive Function	0.017 [-0.708, 0.761]	**-0.375 [-0.697, -0.074]**
Gender – Leaf Fraction	-0.047[-0.239, 0.148]	-0.099[-0.580, 0.394]
Framewise Displacement (Rotation) – Leaf Fraction	-0.074[-0.293, 0.145]	-0.119[-0.456, 0.208]
Framewise Displacement (Translation) – Leaf Fraction	0.048[-0.188, 0.284]	-0.030[-0.356, 0.301]
Factor Loadings		
WMS Digital Span – Working Memory	1.000	1.000
WMS Symbol Span – Working Memory	**0.716 [0.325, 1.235]**	**0.817 [0.546, 1.140]**
WMS Letter Number Sequencing – Working Memory	**0.876 [0.496, 1.386]**	**0.907 [0.656, 1.232]**
D-KEFS Verbal Fluency – Executive Function	1.000	1.000

CPT-D Prime – Executive Function	0.378 [-0.294, 1.221]	0.388 [-0.031, 0.961]
Stroop Conflict Effect – Executive Function	0.294 [-0.250, 0.968]	0.318 [-0.076, 0.851]

Table S5. Model Parameters for model 3.

	Model 3 (n = 40) parameter [95% CI] (PPP = 0.155)
Structural Model Pathways	
Age - Leaf Fraction	**0.407 [0.070, 0.740]**
Leaf Fraction – Negative Symptom	**0.479 [0.194, 0.817]**
Leaf Fraction – Disorganization	0.016 [-0.385, 0.436]
Leaf Fraction – Paranoia	0.156 [-0.260, 0.588]
Age – Negative Symptom	0.023 [-0.253, 0.321]
Age – Disorganization	0.019 [-0.380, 0.410]
Age – Paranoia	0.162 [-0.254, 0.586]
Indirect Effects	
Age – Leaf Fraction – Negative Symptom	**0.184 [0.026, 0.449]**
Age – Leaf Fraction – Disorganization	0.106 [-0.045, 0.378]
Age – Leaf Fraction – Paranoia	0.054 [-0.106, 0.289]
Control Pathways	
Gender – Negative Symptom	-0.230 [-0.836, 0.335]
Gender – Disorganization	0.468 [-0.313, 1.280]
Gender – Paranoia	0.223 [-0.628, 1.078]
Gender – Leaf Fraction	0.849[-0.401, 1.125]
Medication – Negative Symptom	0.113 [-0.155, 0.391]
Medication – Disorganization	0.064 [-0.308, 0.452]
Medication – Paranoia	0.102 [-0.279, 0.495]
Medication – Leaf Fraction	0.294[-0.168, 0.790]
Framewise Displacement (Rotation) – Negative Symptom	-0.164 [-0.565, 0.193]
Framewise Displacement (Translation) – Negative Symptom	0.152 [-0.204, 0.557]
Framewise Displacement (Rotation)	0.004 [-0.495, 0.503]

– Disorganization	
Framewise Displacement (Translation) – Disorganization	0.019 [-0.481, 0.537]
Framewise Displacement (Rotation) – Paranoia	-0.129 [-0.667, 0.407]
Framewise Displacement (Translation) – Paranoia	0.035 [-0.500, 0.567]
Framewise Displacement (Rotation) – Leaf Fraction	-0.360[-0.170, 0.431]
Framewise Displacement (Translation) – Leaf Fraction	-0.115[-1.157,0.814]
Factor Loadings	
Avolition – Negative Symptom	1.000
Attention – Negative Symptom	**0.525 [0.009, 1.176]**
Alogia – Negative Symptom	**0.706 [0.195, 1.462]**
Anhedonia – Negative Symptom	**0.955 [0.521, 1.649]**
Blunt Affect – Negative Symptom	**0.550 [0.040, 1.272]**
Bizarre Behavior - Disorganization	1.000
Positive Formal Thought - Disorganization	0.480 [-0.060, 1.216]
Inappropriate Affect - Disorganization	0.387 [-0.180, 1.185]
Hallucination – Paranoia	1.000
Delusion – Paranoia	**0.571 [0.138, 1.153]**

Table S6. Model Parameters for using a latent variable (network structure) to represent six graph metrics.

	(n = 40) parameter [95% CI] (PPP = 0.098)
Structural Model Pathways	
Age – Network Structure	**0.025 [0.003, 0.062]**
Network Structure– Negative Symptom	**6.281 [1.854, 20.638]**
Network Structure– Disorganization	3.729 [-2.556, 14.864]
Network Structure– Paranoia	1.141 [-6.128, 9.828]
Age – Negative Symptom	0.037 [-0.246, 0.353]
Age – Disorganization	0.034 [-0.367, 0.409]
Age – Paranoia	0.196 [-0.220, 0.620]
Indirect Effects	
Age – Network Structure– Negative Symptom	**0.163 [0.010, 0.422]**
Age – Network Structure– Disorganization	0.084 [-0.060, 0.372]
Age – Network Structure– Paranoia	0.025 [-0.124, 0.225]
Control Pathways	
Gender – Negative Symptom	-0.262 [-0.885, 0.200]
Gender – Disorganization	0.593 [-0.714, 1.475]
Gender – Paranoia	0.127 [-0.567, 0.804]
Gender – Network Structure	0.024[-0.023, 0.078]
Medication – Negative Symptom	0.158 [-0.120, 0.441]
Medication – Disorganization	0.101 [-0.325, 0.487]
Medication – Paranoia	0.112 [-0.266, 0.482]
Medication – Network Structure	0.005[-0.020, 0.033]
Framewise Displacement (Rotation) – Negative Symptom	-0.146 [-0.616, 0.191]
Framewise Displacement (Translation) – Negative Symptom	0.156 [-0.230, 0.551]
Framewise Displacement (Rotation) – Disorganization	-0.009 [-0.538, 0.495]
Framewise Displacement (Translation) – Disorganization	0.024 [-0.452, 0.554]
Framewise Displacement (Rotation) – Paranoia	-0.113 [-0.595, 0.432]

Framewise Displacement (Translation) – Paranoia	0.002 [-0.561, 0.482]
Framewise Displacement (Rotation) – Network Structure	-0.010[-0.052, 0.025]
Framewise Displacement (Translation) – Network Structure	0.000[-0.036, 0.037]
Factor Loadings	
Avolition – Negative Symptom	1.000
Attention – Negative Symptom	**0.537 [0.028, 1.171]**
Alogia – Negative Symptom	**0.691 [0.193, 1.336]**
Anhedonia – Negative Symptom	**0.953 [0.534, 1.636]**
Blunt Affect – Negative Symptom	**0.553 [0.058, 1.278]**
Bizarre Behavior - Disorganization	1.000
Positive Formal Thought - Disorganization	0.480 [-0.004, 1.140]
Inappropriate Affect - Disorganization	0.377 [-0.233, 1.162]
Hallucination – Paranoia	1.000
Delusion – Paranoia	**0.566 [0.139, 1.097]**
Path Length – Network Structure	1.000
Leaf Fraction – Network Structure	**13.150 [7.760 42.016]**
Tree Hierarchy – Network Structure	**7.799 [3.318 28.202]**
Maximum Degree – Network Structure	1.683 [-3.837 9.679]
Assortativity – Network Structure	**-5.559 [-20.606 -1.266]**
Degree Divergence – Network Structure	**11.769 [7.113 38.128]**

Figures

Figure S1. Population distribution in different age group.

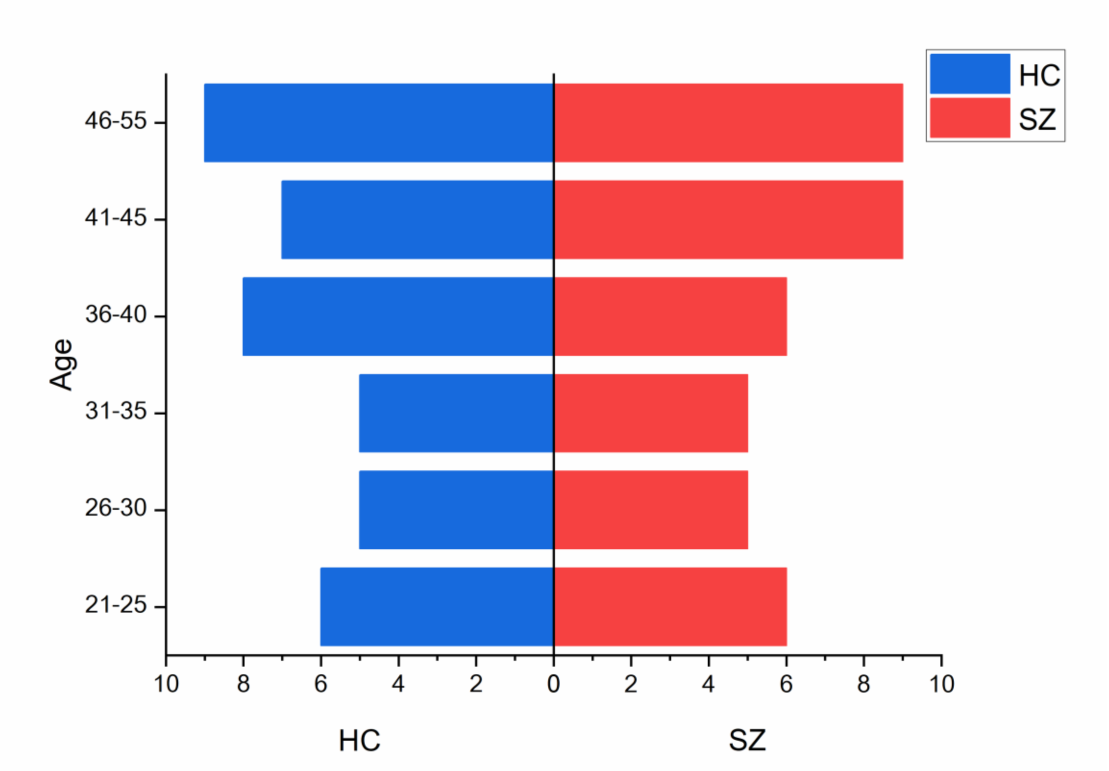

Figure S2. Comparisons of tree metrics with Power's 264-ROI atlas. * indicates significant difference (corrected p<0.05 for 5, 000 permutations), ** for p < 0.01.

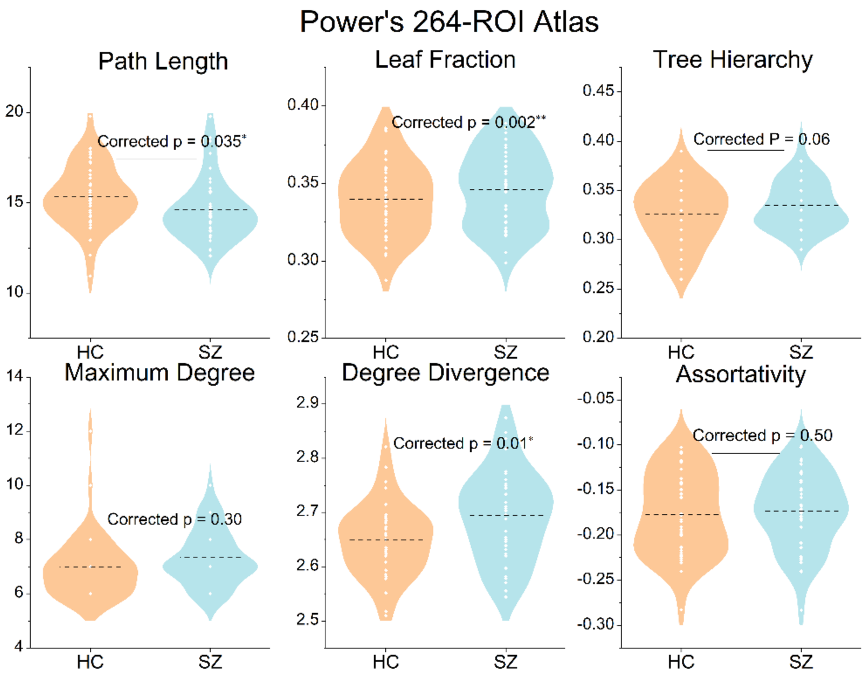

Figure S3. Comparisons of tree metrics with Harvard-Oxford 112-ROI atlas. * indicates significant difference (corrected p<0.05 for 5, 000 permutations), ** for p < 0.01.

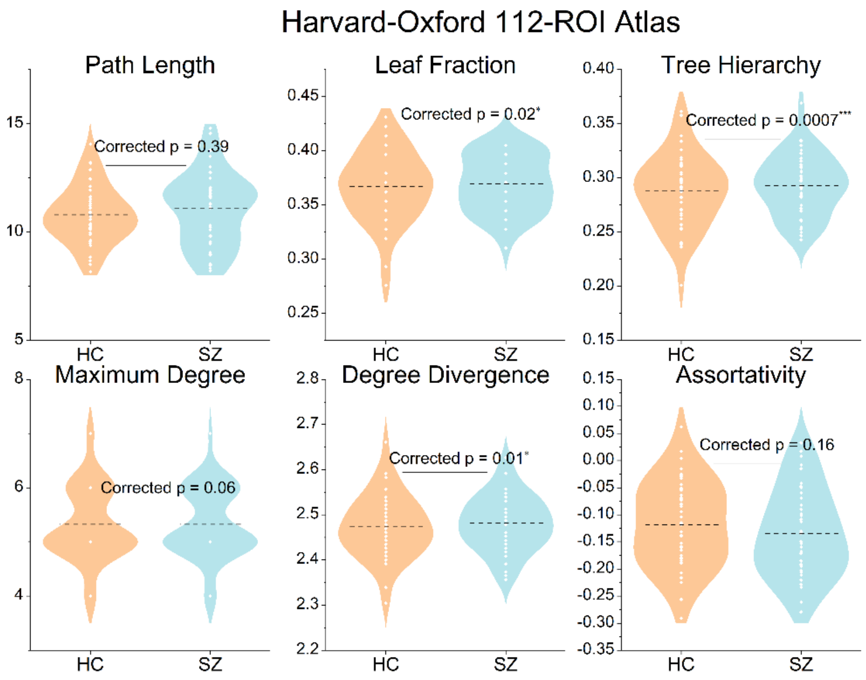

Figure S4. Comparisons of tree metrics with Schaefer 400-ROI atlas. * indicates significant difference (corrected p<0.05 for 5, 000 permutations), ** for p < 0.01.

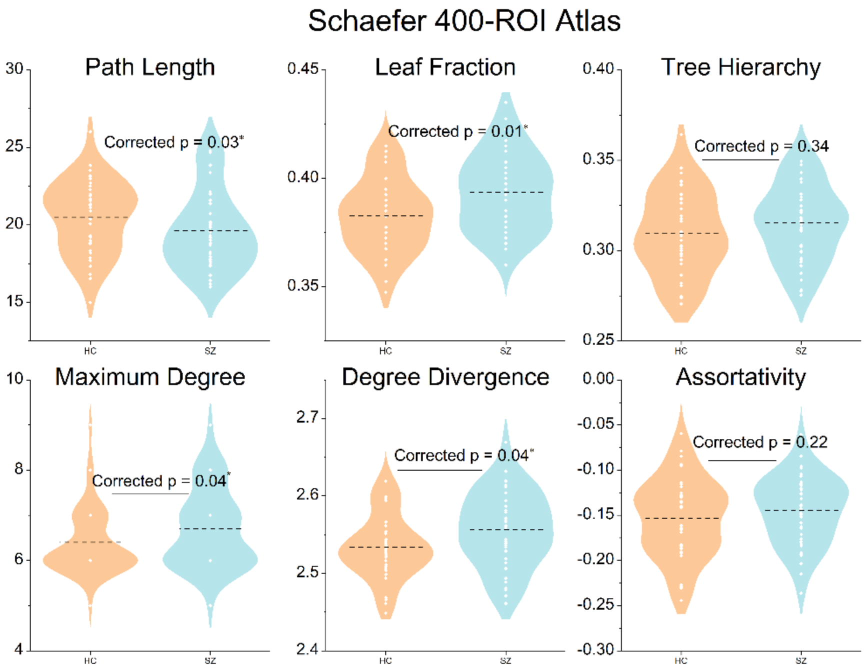

Figure S5. Comparisons of tree metrics with Dosenbach 164-ROI atlas (with additional motion control). * indicates significant difference (corrected p<0.05 for 5, 000 permutations), ** for p < 0.01.

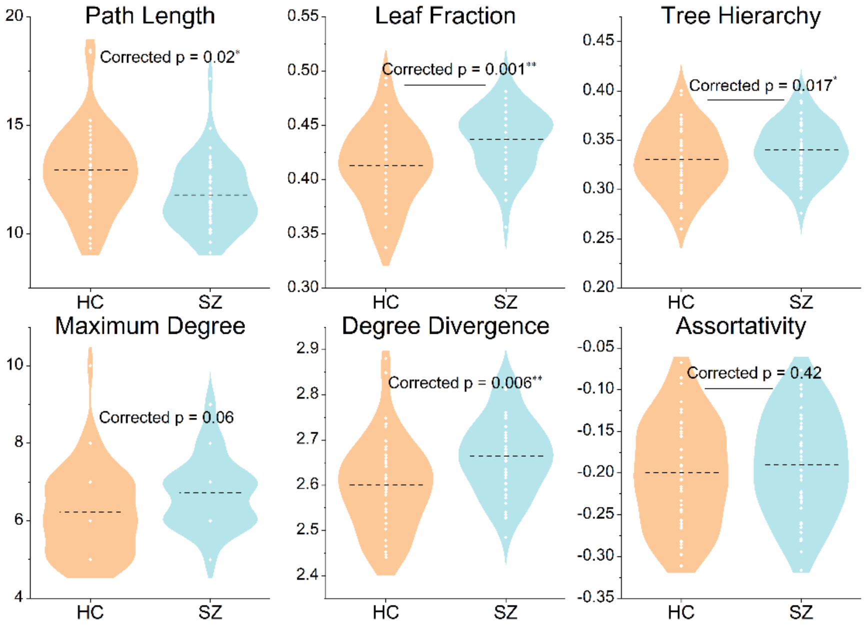

Figure S6. Comparison of tree metrics with removal of four time points. * indicates significant difference (corrected p<0.05 for 5, 000 permutations), ** for p < 0.01.

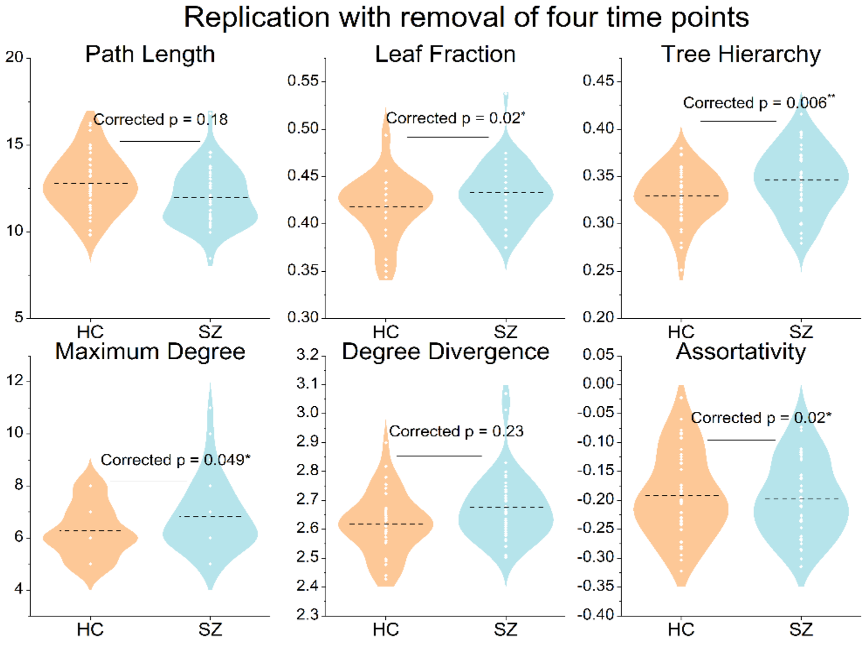

Figure S7. Comparison of tree metrics with global signal regression. * indicates significant difference (corrected p<0.05 for 5, 000 permutations), ** for p < 0.01.

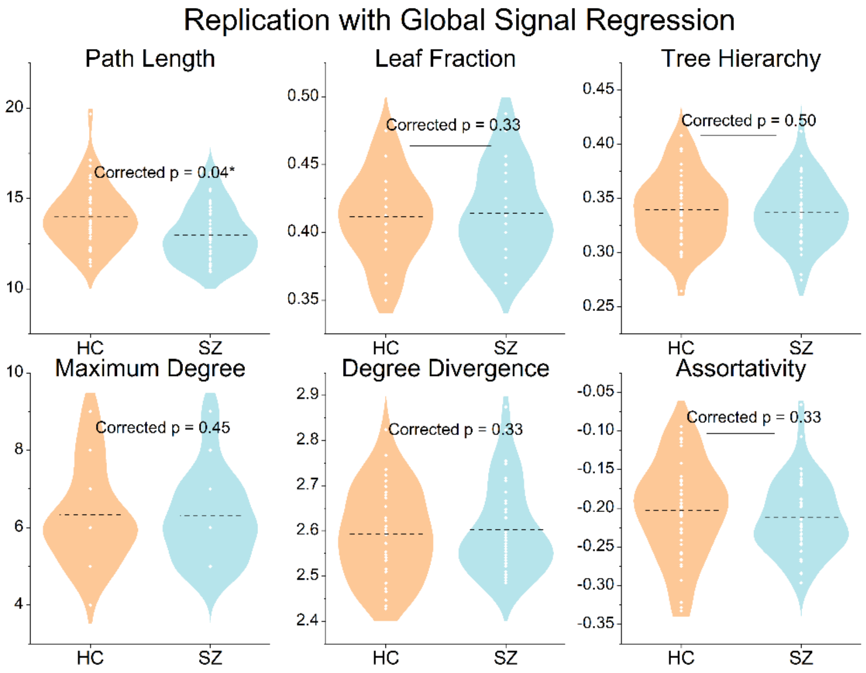

Figure S8. Percentage of negative edges for each subject.

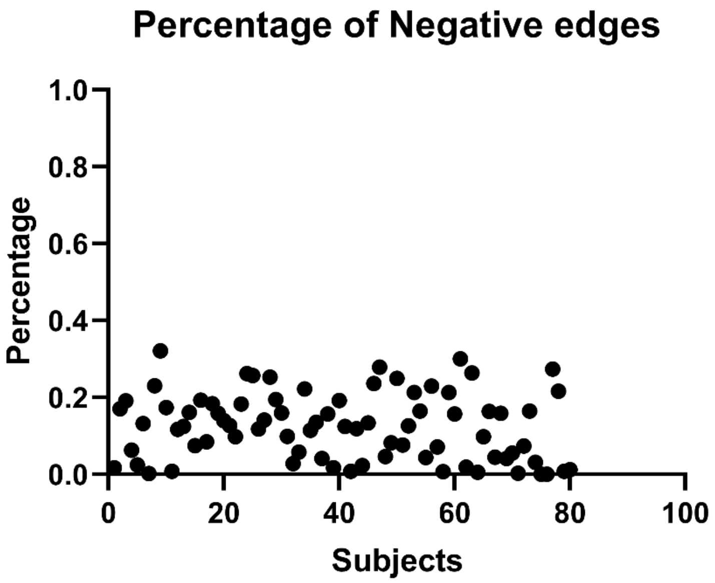

Figure S9. Illustration of model 1 and 2 with covariates and manifest variables. The main part of the model depicts a mediation relationship where leaf fraction (representing brain network structure) mediates the influence of age on behavior (working memory and executive function). The two scores of behavior are obtained by a factor analysis of several behavior measurements. In addition, gender and head motion are controlled as covariates by including them as exogenous predictors of mediator and outcome variables.

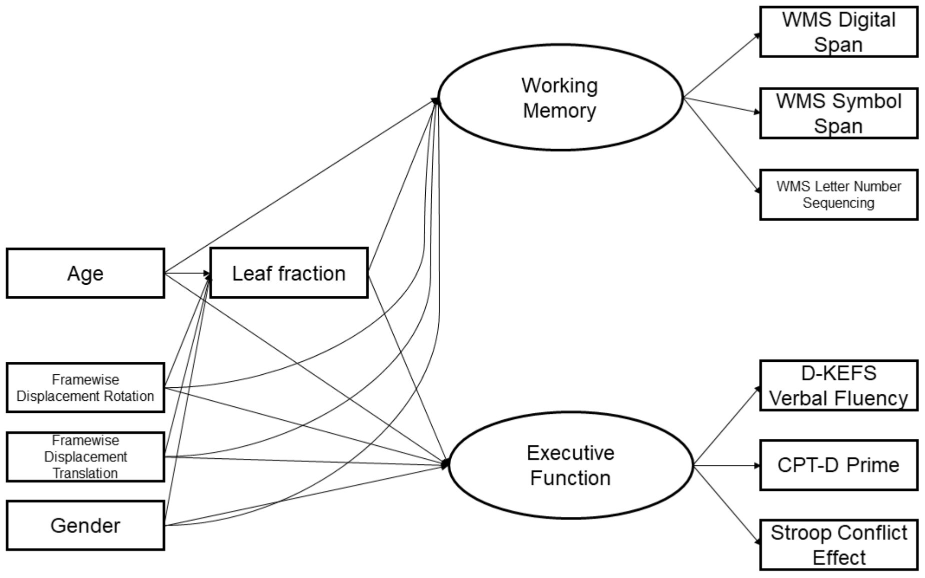

Figure S10. Illustration of model 3. This model is similar to model 1 and 2 but with schizophrenic symptoms instead of cognitive functions as outcome variables. The three dimensions of symptoms (negative symptom, paranoia, disorganization) are obtained from a factor analysis based on ten SANS and SAPS scores.

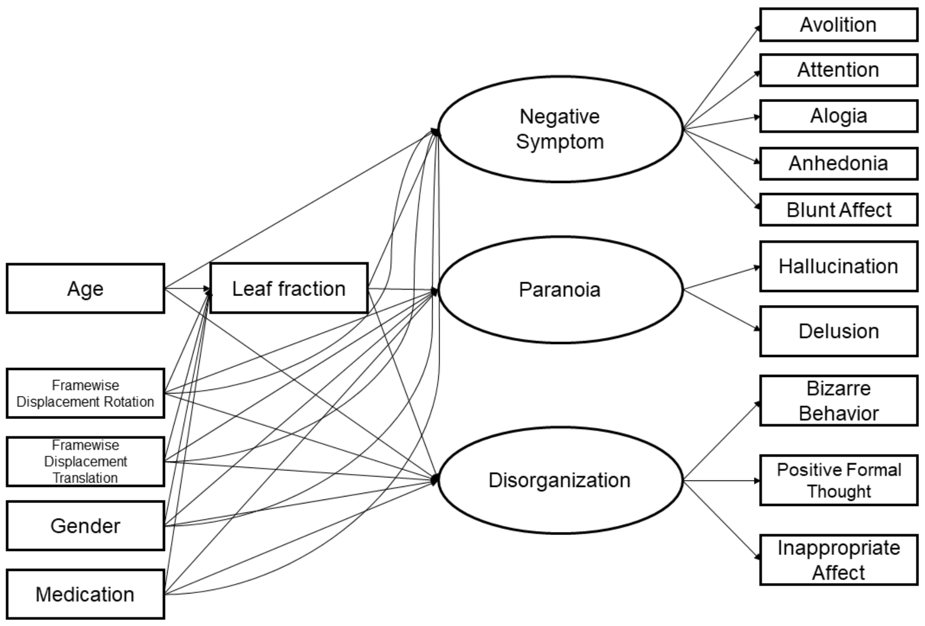

References

Andreasen, N. C., Arndt, S., Alliger, R., Miller, D., & Flaum, M. (1995). Symptoms of Schizophrenia Methods, Meanings, and Mechanisms. *Arch Gen Psychiatry*, *52*(5), 341–351. https://doi.org/10.1001/archpsyc.1995.03950170015003

Chen, X., Liao, X., Dai, Z., Lin, Q., Wang, Z., Li, K., & He, Y. (2018). Topological analyses of functional connectomics: A crucial role of global signal removal, brain parcellation, and null models. *Human Brain Mapping*, *39*(11), 4545–4564. https://doi.org/10.1002/hbm.24305

Kebets, V., Holmes, A. J., Orban, C., Tang, S., Li, J., Sun, N., … Yeo, B. T. T. (2019). Somatosensory-Motor Dysconnectivity Spans Multiple Transdiagnostic Dimensions of Psychopathology. *Biological Psychiatry*, *86*(10), 779–791. https://doi.org/10.1016/j.biopsych.2019.06.013

Kroken, R. A., Johnsen, E., Ruud, T., Wentzel-Larsen, T., & Jørgensen, H. A. (2009). Treatment of schizophrenia with antipsychotics in Norwegian emergency wards, a cross-sectional national study. *BMC Psychiatry*, *9*, 1–9. https://doi.org/10.1186/1471-244X-9-24

Leucht, S., Samara, M., Heres, S., Patel, M. X., Woods, S. W., & Davis, J. M. (2014). Dose equivalents for second-generation antipsychotics: The minimum effective dose method. *Schizophrenia Bulletin*, *40*(2), 314–326. https://doi.org/10.1093/schbul/sbu001

Murphy, K., Birn, R. M., Handwerker, D. A., Jones, T. B., & Bandettini, P. A. (2009). The impact of global signal regression on resting state correlations: Are anti-correlated networks introduced? *NeuroImage*, *44*(3), 893–905. https://doi.org/10.1016/j.neuroimage.2008.09.036

Power, J. D., Cohen, A. L., Nelson, S. M., Wig, G. S., Barnes, K. A., Church, J. A., … Petersen, S. E. (2011). Functional Network Organization of the Human Brain. *Neuron*, *72*(4), 665–678. https://doi.org/10.1016/j.neuron.2011.09.006

Schaefer, A., Kong, R., Gordon, E. M., Laumann, T. O., Zuo, X.-N., Holmes, A. J., … Yeo, B. T. T. (2018). Local-Global Parcellation of the Human Cerebral Cortex from Intrinsic Functional Connectivity MRI. *Cerebral Cortex*, *28*(9), 3095–3114. https://doi.org/10.1093/cercor/bhx179

Tzourio-Mazoyer, N., Landeau, B., Papathanassiou, D., Crivello, F., Etard, O., Delcroix, N., … Joliot, M. (2002). Automated anatomical labeling of activations in SPM using a macroscopic anatomical parcellation of the MNI MRI single-subject brain. *NeuroImage*, *15*(1), 273–289. https://doi.org/10.1006/nimg.2001.0978

Yang, G. J., Murray, J. D., Repovs, G., Cole, M. W., Savic, A., Glasser, M. F., … Anticevic, A. (2014). Altered global brain signal in schizophrenia. *Proceedings of the National Academy of Sciences of the United States of America*, *111*(20), 7438–7443. https://doi.org/10.1073/pnas.1405289111
